# Sulfide Toxicity as Key Control on Anaerobic Oxidation of Methane in Eutrophic Coastal Sediments

**DOI:** 10.1021/acs.est.3c10418

**Published:** 2024-06-18

**Authors:** Paula Dalcin Martins, João P. R. C. de Monlevad, Maider J. Echeveste Medrano, Wytze Klaas Lenstra, Anna Julia Wallenius, Martijn Hermans, Caroline P. Slomp, Cornelia Ulrike Welte, Mike S. M. Jetten, Niels A. G. M. van Helmond

**Affiliations:** † Department of Microbiology, Radboud Institute for Biological and Environmental Sciences, Radboud University, Nijmegen 6525 AJ, The Netherlands; ‡ Department of Ecosystem and Landscape Dynamics, Institute for Biodiversity and Ecosystem Dynamics (IBED), University of Amsterdam, Amsterdam 1098 XH, The Netherlands; § Department of Earth Sciences—Geochemistry, Utrecht University, Utrecht 3584 CB, The Netherlands; ∥ Baltic Sea Centre, Stockholm University, Stockholm 114 18, Sweden

**Keywords:** anaerobic oxidation of methane, coastal sediments, euxinia, eutrophication

## Abstract

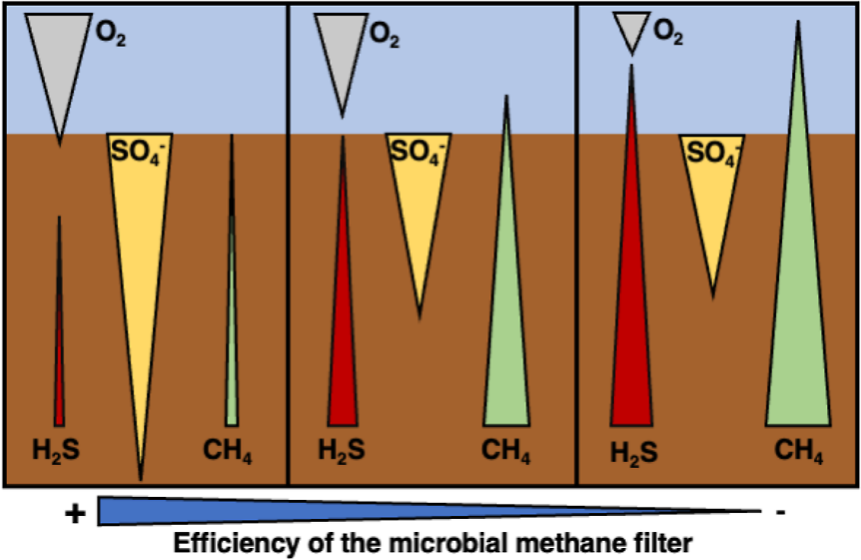

Coastal zones account for 75% of marine methane emissions, despite covering only 15% of the ocean surface area. In these ecosystems, the tight balance between methane production and oxidation in sediments prevents most methane from escaping into seawater. However, anthropogenic activities could disrupt this balance, leading to an increased methane escape from coastal sediments. To quantify and unravel potential mechanisms underlying this disruption, we used a suite of biogeochemical and microbiological analyses to investigate the impact of anthropogenically induced redox shifts on methane cycling in sediments from three sites with contrasting bottom water redox conditions (oxic-hypoxic-euxinic) in the eutrophic Stockholm Archipelago. Our results indicate that the methane production potential increased under hypoxia and euxinia, while anaerobic oxidation of methane was disrupted under euxinia. Experimental, genomic, and biogeochemical data suggest that the virtual disappearance of methane-oxidizing archaea at the euxinic site occurred due to sulfide toxicity. This could explain a near 7-fold increase in the extent of escape of benthic methane at the euxinic site relative to the hypoxic one. In conclusion, these insights reveal how the development of euxinia could disrupt the coastal methane biofilter, potentially leading to increased methane emissions from coastal zones.

## Abstract 2

Factors that control methane escape from coastal ecosystems remain elusive. Here, we present evidence that sulfide is a key factor, being toxic to microorganisms that form the benthic methane biofilter.

## Introduction

Methanogens in marine sediments produce up to 85–300 Tg of the potent greenhouse gas methane per year, which represents 7–25% of global methane production.^1,2^ However, anaerobic methane-oxidizing (ANME) archaea consume more than 90% of the in situ-generated methane.^1^ While coastal zones cover only ca. 15% of the total ocean surface area, they account for more than 75% of global marine methane emissions^3,4^ as an indirect result of high nutrient inputs and burial rates of organic matter.^5^ Recent estimates suggest that 5–28 Tg of methane per year is emitted from coastal waters to the atmosphere.^6^ Eutrophication—excess nutrient input—can further disrupt the balance between microbial methane production and consumption in the (near) future. For instance, eutrophication can cause seawater oxygen depletion due to aerobic microbial respiration coupled to degradation of fresh labile organic matter inputs from increased primary productivity, particularly in enclosed basins with a shallow water depth.^7^ Such oxygen loss is anthropogenically induced in coastal ecosystems^5,8^ and could lead to decreased aerobic methane oxidation in the water column, increasing net methane emissions.^9,10^ Moreover, eutrophication alters sediment geochemistry, such as the vertical compression of the typical marine sedimentary redox zonation,^11^ that could lead to the release of sulfide into bottom waters,^12^ hence the development of euxinia. Additionally, high inputs of organic matter, following eutrophication, provide increased substrates for methanogenesis.^5^ While the combination of these processes is expected to increase methane production in sediments, it remains largely unknown how such processes impact anaerobic methane removal and benthic methane release into the water column. This makes it urgent to mechanistically understand coastal sediment methane dynamics in order to build predictive biogeochemical models of future changes,^13^ develop appropriate management strategies, and accelerate the pace of climate action.

ANME archaea are key players in anaerobic methane removal in marine sediment ecosystems ranging from coastal zones to the deep sea.^14−18^ Phylogenetically, ANME forms three major clades: ANME-1, ANME-2, and ANME-3, assigned a putative nomenclature at family and genus level,^19^ to which we refer in this section. ANME-1, in the order *Methanophagales*, comprises the family *Methanophagaceae* with at least 6 genera, and are present at a broad range of temperatures, from 2 to 100 °C.^20^ Members of this clade have been implicated in both methanogenesis and methane oxidation in estuarine sediments.^21^ ANME-2, in the order *Methanosarcinales*, comprises the family *Methanocomedenaceae*, with two genera, *Ca. Methanocomedens* (ANME-2a) and *Ca. Methanomarinus* (ANME-2b), the family *Methanogasteraceae* (ANME-2c), and the family *Methanoperedenaceae* (ANME-2d). Members of this clade inhabit a narrower range of temperatures (4 to 20 °C).^20^ Finally, ANME-3, also in the order *Methanosarcinales*, comprises the family *Methanosarcinaceae* with the genus *Ca. Methanovorans*, and has been reported in colder temperatures (−1 to 17 °C).^20^ While ANME-1, ANME-2, and ANME-3 have been implicated in sulfate-dependent anaerobic oxidation of methane (S-AOM) in consortia with a syntrophic sulfate-reducing partner,^20,22^ ANME-2d can independently perform nitrate-dependent, iron-dependent, and manganese-dependent anaerobic oxidation of methane (N-AOM, Fe-AOM, and Mn-AOM, respectively).^23−26^ ANME-2a were enriched in Fe-AOM incubations with sediments of the North Sea^14^ and their 16S rRNA gene-based abundance correlated to methane and Fe concentrations in sediments of the Bothnian Sea.^27^ Moreover, ANME-2a, 2b, 2c, 2d and ANME-3 genomes have genes predicted to encode multiheme *c*-type cytochromes potentially implicated in extracellular electron transfer and Fe reduction,^19^ suggesting that multiple ANME groups might perform metal-AOM.

The Baltic Sea is highly impacted by eutrophication^8,28^ and has been proposed as a model marine ecosystem indicative of future global changes related to anthropogenic impacts such as oxygen depletion and environmental degradation.^29^ High methane emissions to the atmosphere from several locations in the Baltic Sea have been documented, in the range of 0.1–3.3 mmol m^–2^ day^–1^, particularly from coastal sites with a shallow sulfate–methane transition zone (SMTZ) in the sediment and relatively shallow water depth.^3,30,31^ Similarly, significant methane concentrations in the water column (up to 47 nmol L^–1^),^31^ large benthic fluxes of methane (up to 2.6 mmol m^–2^ day^–1^),^32^ and high porewater concentrations of methane (6 mM)^32^ have been reported in the Baltic Sea. Previous studies indicate that ANME are key players in methane cycling in Baltic Sea sediments, with ANME-1 and ANME-2 accounting for S-AOM activity, ANME-2 potentially involved in Fe-AOM, and ANME-1 also implicated in methanogenesis.^27,33−35^ However, a mechanistic understanding of the environmental and biological factors that impact AOM in the Baltic Sea and other coastal ecosystems remains elusive.

Here, we investigated sediments of the eutrophic Stockholm Archipelago,^8,36,37^ pursuing a better mechanistic understanding of the impacts of divergent bottom water redox conditions on microbial methane cycling and associated sediment biogeochemistry. We selected three sites with contrasting bottom water oxygen concentrations (oxic: [O_2_]_aq_ > 63 μmol L^–1^; seasonally hypoxic: [O_2_]_aq_ < 63 μmol L^–1^; euxinic: [O_2_]_aq_ = 0 μmol L^–1^ with free sulfide). Sediment cores taken at these sites were subjected to high-resolution geochemical characterization, 16S rRNA gene profiling, potential methane production rate measurements, metagenomic analyses, and AOM rate measurements in selected sediments incubated with different sulfide concentrations. Our study specifically aimed to identify the main controls on the abundance, distribution, and activity of ANME archaea and to elucidate the impacts of differing bottom water redox conditions on methane cycling in these eutrophic coastal sediments.

## Materials and Methods

### Sampling

**Figure 1:**
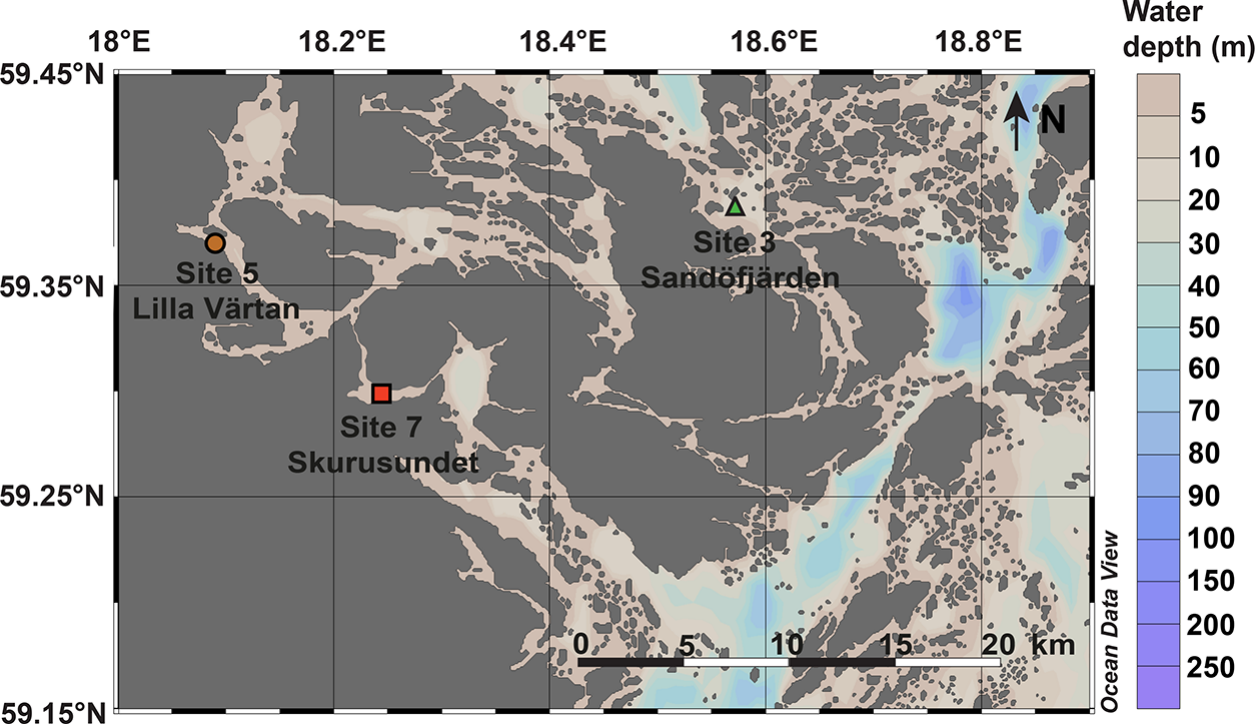
Sampling locations within the Stockholm Archipelago selected for this study. Bottom water redox conditions at each site are color coded with a green triangle (oxic), an orange circle (hypoxic), and a red square (euxinic). This figure was generated using Ocean Data View^38^ with permission (changes were made) from Copyright 2023 Ocean Data View https://creativecommons.org/licenses/by/4.0/.

Sampling was carried out on board the R/V *Electra* from June 11 to 13, 2019, at three locations in the Stockholm Archipelago with contrasting bottom water redox conditions, that were selected based on extensive environmental monitoring by the Swedish Meteorological and Hydrological Institute (SMHI; https://sharkweb.smhi.se/hamta-data/): Site 3 (Sandöfjarden — oxic), Site 5 (Lilla Värtan — seasonally hypoxic), and Site 7 (Skurusundet — euxinic) (Figure 1). Prior to sediment retrieval, a CTD instrument (SBE 911plus, Sea-bird Scientific, USA) was deployed to determine in situ water column characteristics, such as dissolved oxygen concentrations, temperature, and salinity. Site coordinates and characteristics are provided in Tables 1 and 2. At each study site, nine sediment cores were collected with a Gemini gravity corer using transparent PVC core liners of 80 cm in length and an inner diameter of 8 cm. Three cores were sieved over a 0.5 mm mesh size, after which macrofauna was determined at the genus level. One core was transferred to the onboard laboratory for high-resolution depth profiling of oxygen, pH, and sulfide using microelectrodes. Samples for bottom and porewater methane were collected from another core directly after its retrieval with cutoff syringes via predrilled holes covered with tape prior to coring. One core was sliced in a glovebag under a nitrogen atmosphere. First, two bottom water samples were taken from the overlying water, after which sediment slices were collected in 50 mL conical tubes, centrifuged, and subsampled for a range of solutes. One core was sliced under ambient atmospheric conditions to determine the water content of the sediment. Details on core handling, resolution, and porewater extraction are described in the supplement. The last two cores were sliced under a nitrogen atmosphere for microbiological analyses, as detailed below.

**Table 1:** Geographical Coordinates and Characteristics of Each Sampling Site

site	coordinates (DD°MM′SS″)	water depth (m)^a^	bottom water redox conditions^a^	location in the Archipelago^b^
site 3 (Sandöfjarden)	59°24′33″N 18°31′14″E	64	oxic	intermediate
site 5 (Lilla Värtan)	59°22′08″N 18°05′35″E	21	seasonally hypoxic	inner
site 7 (Skurusundet)	59°17′55″N 18°13′46″E	27	generally euxinic	intermediate

a Based on monitoring data from the Swedish Meteorological and Hydrological Institute in 2019 (https://sharkweb.smhi.se/hamta-data/).

b Following a previously described classification system.^37^

**Table 2:** Characterization of Sampling Sites^a^

site	oxygen (μmol L^–1^) and pH at sediment–water interface^b^	oxygen penetration depth (mm)^b^	BW salinity	BW temperature (°C)	macrofauna	average TOC (%)^b^
site 3 (Sandöfjarden)	150 7.4	5.3	5.8	2.1	*Marenzelleria* sp. and *M. balthica*	5.1
site 5 (Lilla Värtan)	55 7.3	1.4	4.9	2.3	none	5.9
site 7 (Skurusundet)	0^c^ 7.1	0	5.3	2.8	none	8.5

a BW, bottom water; TOC, total organic carbon.

b Full profiles in Table S3.

c At Site 7, sulfide (10 μmol L^–1^) was present in the bottom water.

### Porewater Analysis

Methane concentrations were determined with a gas chromatograph equipped with a flame ionization detector after addition of nitrogen headspace of 10 mL and equilibration of the gas and water phase for a week.^36^ Porewater sulfide and ammonium concentrations were measured spectrophotometrically with phenylenediamine and ferric chloride^39^ and indophenol-blue,^40^ respectively. Iron and Mn were measured by inductively coupled plasma-optimal emission spectroscopy (ICP-OES), and nitrate and nitrite were determined with a discrete analyzer and sulfate by ion chromatography. Details are described in the supplement.

### Flux Estimates

The downward and upward fluxes of sulfate and methane, respectively, into the SMTZ and the diffusive fluxes of methane across the sediment–water interface were calculated using Fick’s first law of diffusion
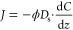
where *J* represents the diffusive flux (mmol m^–2^ d^–1^), ϕ represents the sediment porosity, *D*_s_ represents the sediment diffusion coefficient for the ambient tortuosity, pressure, temperature, and salinity at each site were calculated using the *R* package *marelac*,^41^ which implements the constitutive relations previously listed^42^ and d*C*/d*z* is (1) the concentration gradient of sulfate from above the SMTZ into the SMTZ; (2) the concentration gradient of methane into the SMTZ from below; and (3) the concentration gradient between the top layer of the sediment and the bottom water. We note that the methane fluxes may be underestimated in sediment sections with high methane concentrations because of porewater methane loss due to degassing during sampling, as observed in previous studies.^43,44^ Sediment sulfide exposure was calculated based on sulfide profiles and ^210^Pb-based sedimentation rates (Supporting Information and Table S3).

### Sediment Analysis

Sediment samples from the core that was sliced under a nitrogen atmosphere were freeze-dried, ground, and homogenized in a nitrogen-filled glovebox, using an agate mortar and pestle, and subsequently separated into a fraction that was stored under oxic conditions (oxic fraction) and a fraction that was stored under a nitrogen atmosphere (anoxic fraction). A subsample of the oxic fraction was decalcified, after which organic carbon and nitrogen were measured with an elemental analyzer. The total sedimentary concentrations of Fe and Mn were measured by ICP-OES after triple acid digestion of a subsample of the oxic fraction. To separate the different sedimentary forms of Mn^45^ and Fe,^46^ two previously described protocols were applied on subsamples of the anoxic fraction. Details are described in the supplement.

### DNA Extractions, Amplicon Sequencing, and 16S rRNA Gene Analyses

Sediments for DNA sequencing were immediately frozen at −20 °C after on board core slicing under a nitrogen atmosphere and were stored at −20 °C for 4 months until thawing at room temperature for DNA extractions. DNA was extracted from 73 sediment samples retrieved from three cores in total, one core per site, with a depth resolution of 0.5 cm for the top 2 cm, 1 cm until 10 cm depth, 2 cm until 20 cm depth, and 4 cm below that. DNA extractions were performed with the DNeasy Power Soil Kit (Qiagen, Germany) according to the manufacturer’s instructions with a few modifications (see the Supporting Information). Amplicon sequencing was conducted by Macrogen Europe BV (Amsterdam, Netherlands) on an Illumina MiSeq platform using the archaeal primers Arch349F and Arch806R,^47^ producing 2 × 300 bp in paired-end reads. 16S rRNA gene sequencing data were processed with the DADA2 pipeline^48^ (see details in the Supporting Information).

### Metagenomic Sequencing and Data Analyses

For each site, DNA was extracted from homogenized sediment samples from four intervals: 0–4, 9–12, 21–24, and 33–36 cm. These 12 samples were sequenced by Macrogen Europe BV (Amsterdam, Netherlands) using the TruSeq Nano DNA library with an insert size of 350bp on an Illumina NovaSeq6000 platform, producing 2 × 151 bp paired-end reads. Reads were processed to generate metagenome-assembled genomes (MAGs) as previously described^49^ and as detailed in the supplement. MAGs were taxonomically classified with GTDB-Tk v1^50^ and annotated with DRAM v1.0.^51^ Only high- and medium-quality MAGs (>50% complete and less than 10% contaminated) were included in genome-centric analyses, and the entire data set (binned and unbinned contigs) was considered in gene-centric analyses. For phylogenetic trees, sequences were aligned with muscle v3.8.31,^52^ alignment columns were stripped with trimAl v1.4.rev22,^53^ and trees were built with FastTree v2.1.10^54^ or UBCG v3.0.^55^ Average amino acid identity between selected genomes was calculated using the Konstantinidis Lab tool (http://enve-omics.ce.gatech.edu/g-matrix/index). The abundance of MAGs was inferred from normalized genome coverage (ngCOV), that is, MAG coverage normalized to total metagenome size.^56^ Normalized mapped read (NMR) values for specific genes were calculated as follows: NMR = the number of mapped reads to the gene/(the length of the gene in bp × 10^3^) × (the total number of reads (mapped + unmapped) in the metagenome/10^6^). See the Supporting Information for more details.

### Potential Methane Production Rate Measurements

Sediments for incubations to measure potential methane production rates were sliced in a N_2_-filled portable glovebag and placed into sterile plastic bags (VWR International BV, Amsterdam). The resulting slices had a resolution of 4 cm and were stored anoxically into sealed aluminum bags (Gruber-Folien GmbH & Co. KG, Germany) in the dark at 4 °C for 1 month from sampling collection until bottles were assembled. For this, 5 g of wet sediments was placed into 60 mL-serum bottles, and 5 mL of sulfate-free artificial seawater (ASW) medium at pH 7.5 was added to create a 1:1 diluted slurry. Bottles were degassed and sealed under argon gas at atmospheric pressure (Linde Gas Benelux). The ASW medium was adapted from a previously published study^57^ to achieve a salinity of 5.3^36^ and contained, per liter, 3.418 g NaCl, 1.54 g MgCl_2_·6H_2_O, 0.097 g KCl, 0.21 g CaCl_2_·2H_2_O, 0.014 g KBr, 0.0037H_3_BO_3_, 0.003 g SrCl_2_·6H_2_O. 0.0004 g of NaF, and 0.028 g of NaHCO_3_. No trace elements or vitamins were added. From each site, six depths were incubated in triplicate at 4 °C, resulting in 54 serum bottles. Methane production was monitored via injection of 100 μL-headspace samples into an HP 5890 gas chromatograph equipped with a Porapak Q column (80/100 mesh) and flame ionization detector (Hewlett-Packard, USA) with a detection limit of <1 ppm. Each gas sample was measured in triplicate and areas were averaged. Methane concentrations were calculated using the Henry’s law coefficient H^cp^.^58^ Potential rates of methane production were calculated using a linear regression of methane measurements obtained in the first 8–9 days of sediment incubation. More details are provided in the supplement and all calculations are provided in Table S1.

### Sulfide Toxicity Experiment and AOM Rate Measurements

Sediments for incubations to measure AOM rates under different sulfide concentrations were retrieved from previously stored, anoxically sealed aluminum bags kept in the dark at 4 °C for approximately 2 years after sample collection until bottles were assembled. For these incubations, Site 5 was selected due to the highest methanotroph genome coverages, and samples were combined and homogenized, resulting in a mixture spanning the depth of 8 to 28 cm. Five grams of wet sediments was placed into 60 mL-serum bottles and mixed with 5 mL of a solution containing MgSO_4_ 8 mM and 0.5% NaCl, to achieve a final concentration of 4 mM sulfate and a pH buffered to 7.05. Bottles were degassed with argon gas, and a headspace at 0.5 bar of overpressure was created, containing also 0.5% of N_2_, 0.5% CO_2_) and 20% ^13^CH_4_. To one set of triplicate bottles, sulfide was added to a final concentration of 2 mM, while the other bottles did not receive sulfide. After 13 weeks of incubation in the dark at 4 °C, AOM was confirmed by the detection of ^13^CO_2_ production (data not shown). Then, sulfide was added to the remaining bottles, in triplicate incubations, targeting final concentrations of 0, 0.5, 1, 2, and 4 mM. To monitor ^13^CO_2_ production, bottle pressure was monitored using a GHM 3111 Digital Pressure Meter with a GMSD 2 BR-K31 sensor, and 50 μL of headspace was injected into an Agilent 6890 series gas chromatograph coupled to a mass spectrometer equipped with a Porapak Q column heated at 80 °C with helium as the carrier gas as previously described.^59^ Liquid-dissolved ^13^CO_2_ was estimated with the equation ∑^13^CO_2_ = ^13^CO_2_(g) [1 + *k*RT *V*_liquid_/*V*_gas_ (1 + *K*_Z_/[H^+^])]^60^ and summed to headspace ^13^CO_2_ derived from calibration curves for AOM rate calculations (see Table S2 for calculations). Bottles were sampled for sulfide determination as described in the section on collection of porewater and sediment samples for geochemical analysis. For that, approximately 1 mL of slurry was anoxically withdrawn and filtered through a 0.2 μm Nylon syringe filter. Approximately 300 μL of filtrate was mixed with 1.2 mL of an anoxic 2% zinc acetate solution and stored at 4 °C until analysis. Removed liquid volumes were considered for the AOM rate calculations. Finally, sulfide concentrations were measured as described in the section “Porewater Analysis”.

### Data Availability

Adapter-trimmed 16S rRNA gene reads and MAGs have been deposited with NCBI under BioProject PRJNA805085. All geochemical data in this study are provided in Table S3.

## Results and Discussion

### Sulfate-Dependent Anaerobic Oxidation of Methane Is the Dominating Process for Benthic Methane Removal

At the time of sampling, water column characteristics at the three study sites were coherent with historical environmental monitoring data by SMHI (Tables 1 and 2, and Figure S1).^33^ Oxygen penetration into the sediment followed the trend in ambient bottom water redox conditions, with the deepest oxygen penetration at Site 3 and no oxygen penetration at Site 7 (Table S3). In fact, for the latter, sulfide (10 μmol L^–1^) was detected in the bottom water (Tables 2 and S3). Macrofauna (polychaetes and bivalves) were observed only at Site 3. While bottom water pH, salinity, and temperature were similar across sites, the different bottom water redox conditions were reflected in full profile-averaged sedimentary TOC contents, with the highest TOC observed at the euxinic site, Site 7 (Tables 2 and S3). However, taken together, the three sites are geochemically rather similar and comparable to other sites in the Stockholm Archipelago that were previously geochemically characterized, i.e., organic-rich sediments, sulfide accumulation in the porewaters, and a shallow SMTZ.^32,36^

**Figure 2:**
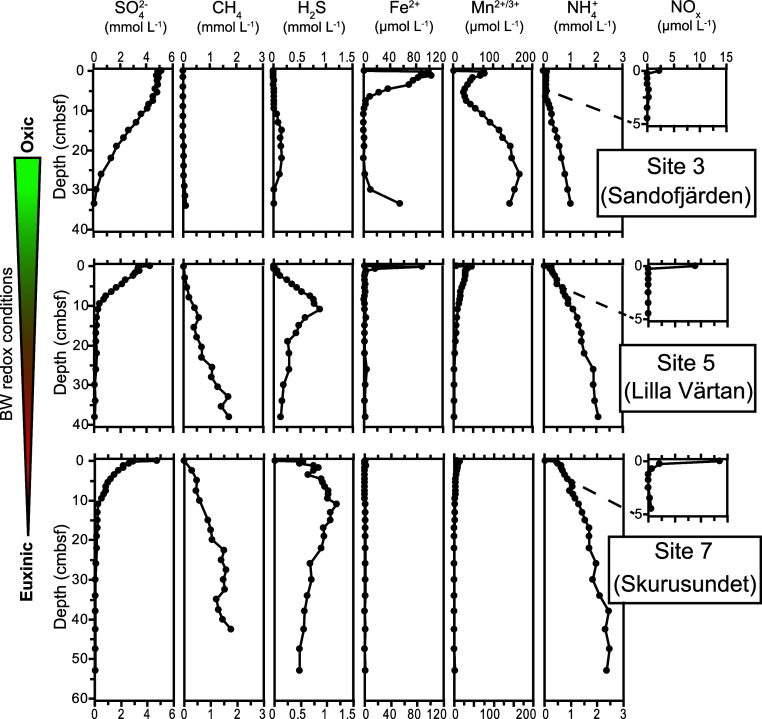
Porewater depth profiles of sulfate (SO_4_^2–^), methane (CH_4_), sulfide (H_2_S), dissolved iron (Fe^2+^) and manganese (Mn^2+/3+^), ammonium (NH_4_^+^), and the sum of nitrate and nitrite (NO_*x*_) at our study sites in the Stockholm Archipelago: Site 3 (Sandofjärden), Site 5 (Lilla Värtan), and Site 7 (Skurusundet). The arrow to the left indicates decreasing bottom water (BW) oxygen concentrations from Site 3 to 7. Cmbsf; centimeters below the seafloor.

The observed shallow SMTZ is a common feature of eutrophic coastal sediments.^5^ Porewater sulfate decreased with depth at all sites (Figure 2 and Table S3). At Site 3, it took ∼30 cm below the seafloor (cmbsf) before sulfate was depleted, whereas at Sites 5 and 7, this removal occurred already around 10 cmbsf (Figure 2). Methane concentrations increased slightly with depth to a maximum of ca. 100 μmol L^–1^ at Site 3. At Sites 5 and 7, in contrast, concentrations of methane increased strongly with depth, reaching a value of ∼2 mmol L^–1^. Porewater sulfide was only present at low concentrations (<200 μmol L^–1^) and in a confined zone (10–30 cmbsf) at Site 3. At Sites 5 and 7, however, sulfide concentrations increased rapidly with depth, with the strongest increase and highest concentrations (>1 mmol L^–1^) at Site 7. Additionally, sediments in Site 7 had a higher approximated annual sulfide exposure (0.88 mmol year^–1^) in comparison to Site 5 (0.39 mmol year^–1^) and Site 3 (0.08 mmol year^–1^) (Table S3). The shallow SMTZ is the combined result of low salinity, hence low sulfate concentrations and high rates of organic matter deposition and degradation, culminating in a vertical compression of the redox zonation.^61^ The somewhat deeper SMTZ at Site 3 can be explained by its ambient redox conditions. Oxygen is perennially available throughout the water column, leading to more aerobic degradation of organic matter in the water column and surface sediments, hence the extended depth of the depletion of alternative electron acceptors, such as sulfate (Figure 2).

At Sites 3 and 5, a peak in dissolved Fe was observed directly below the sediment–water interface, followed by a rapid decrease to values of around zero. At Site 3, dissolved Fe concentrations increased again when sulfide was depleted at a depth. Dissolved Fe was nearly absent at Site 7. A peak in dissolved Mn was observed near the sediment–water interface at all three sites, with maximum concentrations decreasing with increasingly reducing sediments from Site 3 to Site 5 to Site 7. At Site 3, dissolved Mn in the porewater increased again at a depth after a subsurface minimum. Ammonium increased with depth at all sites, with the highest concentrations (up to 2.5 mmol L^–1^) and most rapid increase at Sites 5 and 7. NO_*x*_ (nitrate and nitrite) concentrations ranged from 2.5 to 12.5 μmol L^–1^ in the bottom waters and decreased rapidly with depth in the sediment at all sites (Figure 2 and Table S3).

The calculated downward flux of sulfate into the SMTZ (Table 3) was highest at Sites 5 and 7 (1.5 and 1.3 mmol m^–2^ d^–1^), but still substantial (0.9 mmol m^–2^ d^–1^) at Site 3. The calculated upward flux of methane into the SMTZ at Site 3 was nearly absent, and around 0.5 mmol m^–2^ d^–1^ at Sites 5 and 7, which should be regarded as an absolute minimum estimate due to degassing of methane during sampling.^43,44^ There was no benthic methane efflux at Site 3. The benthic methane efflux was about seven times larger at Site 7 relative to Site 5 (∼1 vs 0.15 mmol m^–2^ d^–1^, respectively).

**Table 3:** Diffusive Fluxes of Sulfate and Methane (mmol m^–2^ day^–1^) as Calculated from Porewater Profiles (Intervals in Parentheses) and, for the Benthic Flux, from Porewater and Bottom Water Concentrations (See Text)^a^

site	downward sulfate flux into SMTZ	upward methane flux into SMTZ^b^	benthic methane efflux
site 3 (Sandöfjarden)	0.88 (9.5–19 cm)	0.08 (29–34 cm)	0
site 5 (Lilla Värtan)	1.49 (1.75–7.5 cm)	0.54 (8–13 cm)	0.15
site 7 (Skurusundet)	1.27 (2.5–4.5 cm)	0.50 (0–15 cm)	1.02

a SMTZ; sulfate–methane transition zone.

b Methane fluxes into the SMTZ at Sites 5 and 7 are likely underestimated because of degassing during sampling.

In sediments of eutrophic, low-oxygen coastal systems, S-AOM is expected to dominate methane removal.^1^ Sulfate is presumably also the major terminal electron acceptor for AOM by ANME-2 archaea in the investigated sediments of the Stockholm Archipelago at all three sites. Estimated diffusive fluxes of sulfate and methane into the SMTZ (Table 3) suggest that S-AOM accounts for at least 40% of the observed sulfate reduction. Given the potential degassing of methane, especially for sediment intervals with high methane concentrations,^44^ in situ S-AOM rates are likely higher. The differences in calculated benthic effluxes of methane between our three sites suggest that the removal of methane becomes less effective with more reducing bottom water redox conditions (Table 3).

**Figure 3:**
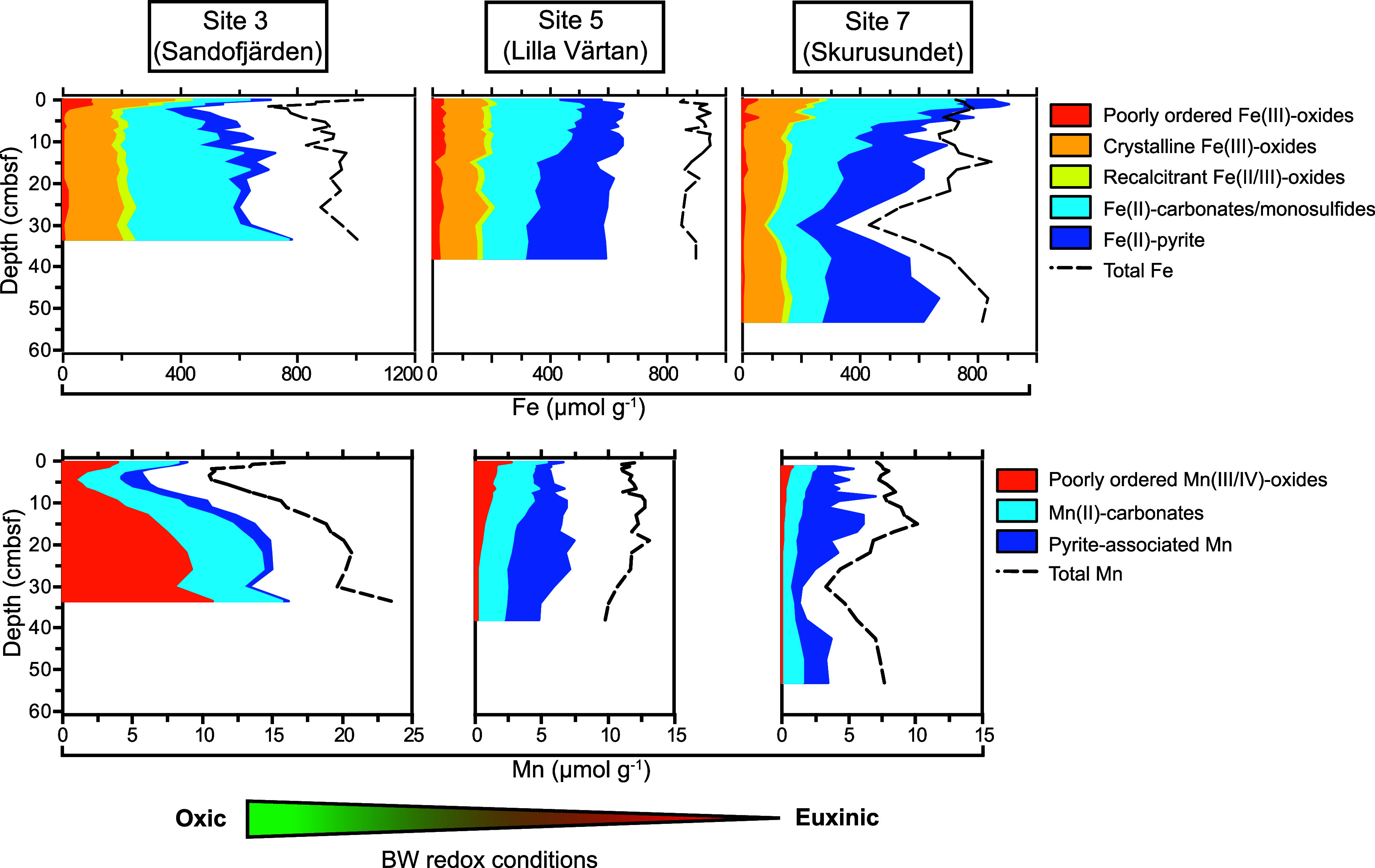
Solid-phase iron and manganese speciation (μmol g^–1^ dry sediment) depth profiles for the three study sites. The arrow at the bottom indicates decreasing bottom water (BW) oxygen concentrations from Site 3 to 7. cmbsf; centimeters below the seafloor.

To investigate if alternative terminal electron acceptors for AOM such as Fe and Mn oxides were available, we determined the concentration and reactivity of the Fe and Mn oxides present in the sediment. The sequential extractions dissolved between 70 and 80% of the total sediment Fe at the three sites (Figure 3 and Table S3). Except for the surface sediment at Site 3, the poorly ordered Fe(III)-oxides (e.g., ferrihydrite and lepidocrocite) content was generally low. Crystalline Fe(III)-oxides (e.g., goethite and hematite) accounted for a substantial fraction of the extractable Fe at all sites (15–30%). The fraction consisting of a mixture of Fe(II)-carbonates and monosulfides, merely consisting of Fe(II)-monosulfides in this setting with abundant porewater sulfide,^36^ was the dominant Fe fraction at depth in the sediment at Site 3. At Sites 5 and 7, this fraction, as well as Fe(II)-pyrite, accounted for the majority of the extractable Fe at depth.

About 50 to 60% of the total sedimentary Mn was extracted in the analyzed steps, i.e., steps 1, 2, and 5 (see the Supporting Information section on Solid-Phase Analysis), at all three sites. At Site 3, poorly ordered Mn(III/IV)-oxides (e.g., birnessite and pyrolusite) dominated the extracted Mn pool with only a minor (<10%) contribution of pyrite-associated Mn. At Site 5, poorly ordered Mn(III/IV)-oxides were never dominant, and concentrations were low at depth. Sedimentary Mn could generally be divided into two relatively equal fractions of Mn(II)-carbonates (rhodochrosite) and pyrite-associated Mn. At Site 7, pyrite-associated Mn dominated the extracted Mn pool with only a minor (<10%) contribution of poorly ordered Mn(III/IV)-oxides (Figure 3 and Table S3).

In coastal systems, electron acceptors other than sulfate may drive AOM, such as nitrate and nitrite^23,60^ as well as poorly ordered Fe(III)- and Mn(III/IV)-oxides.^14,61,62^ At our sites, nitrate and nitrite are exclusively present in low concentrations in the surface sediment (Figure 2) and are therefore unlikely to substantially contribute to AOM activity. At Sites 5 and 7, poorly ordered Fe(III)- and Mn(III/IV)-oxides are nearly absent (Figure 3). Additionally, at both these sites, most of the reactive Fe and Mn is sulfidized (Figure 3), in line with the ambient bottom water redox conditions and relatively high porewater sulfide concentrations (Table 2 and Figure 2), previously also observed for other sites in the Stockholm Archipelago.^36^ Hence, there is only limited potential for Fe- and Mn-AOM, which seem to play a larger role in oligotrophic rather than eutrophic coastal ecosystems.^63−65^ However, a role for Fe-AOM cannot be fully excluded, as crystalline Fe(III)-oxides and even recalcitrant Fe(II/III)-oxides (Figure 3) may also play a role in Fe-AOM^66^ and S-AOM.^67^ Magnetite, for example, was shown to stimulate Fe-AOM activity and ANME-2a enrichment in incubations with North Sea sediments,^14^ and goethite-dependent AOM has been suggested as a significant methane sink in paddy soils, in which hematite and magnetite-AOM were also detected.^68^

### High Methane Production Potential in the Hypoxic and Euxinic Sites

**Figure 4:**
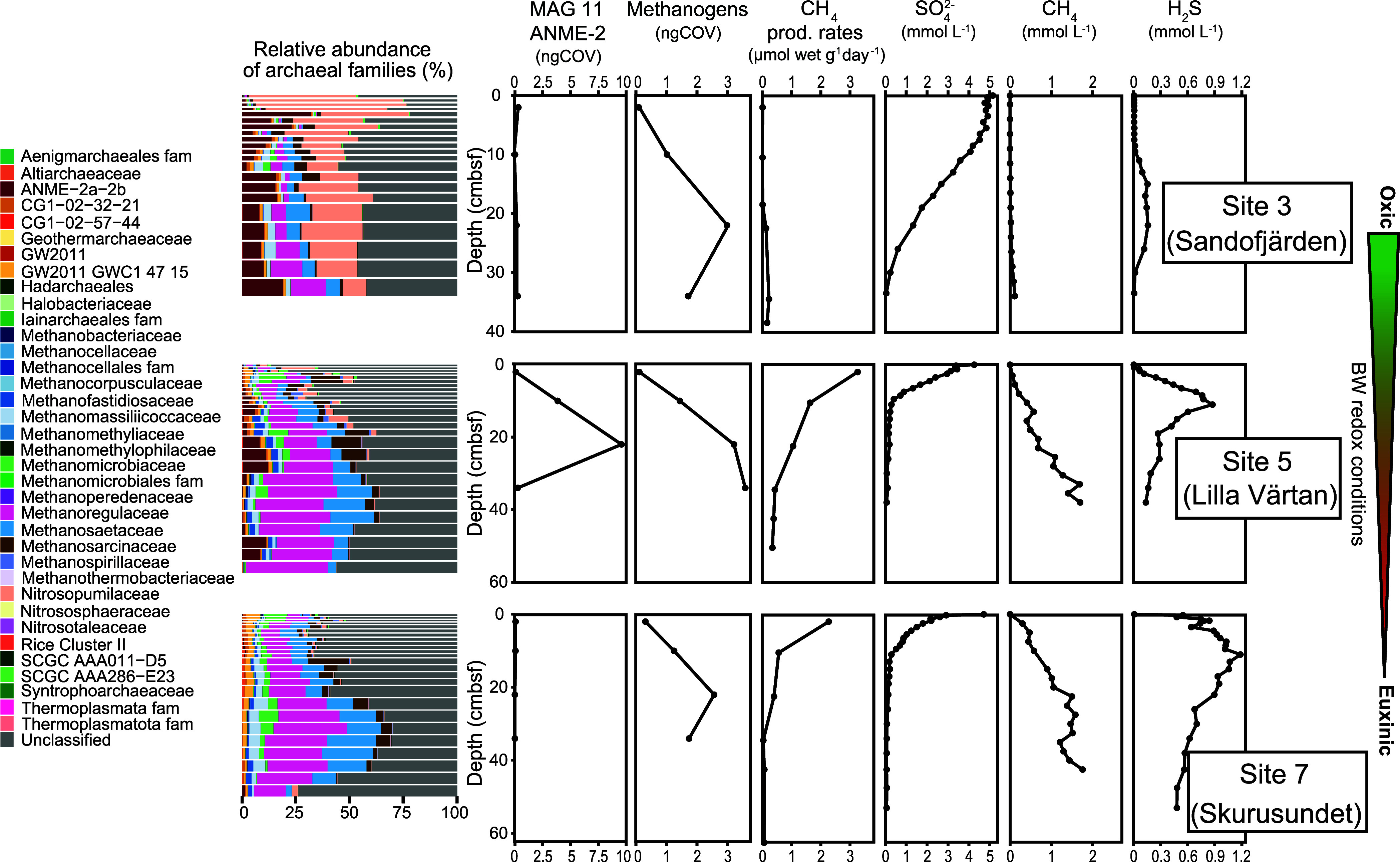
Abundance, distribution, and activity of key microbial groups in sediments from the three study sites presented with selected geochemical data. Relative abundances (%) of archaeal families, based on 16S rRNA gene sequencing, are color coded according to the legend to the left and match depths as in other panels. The designation “fam” indicates a poorly resolved family. The thickness of bars corresponds to the depth resolution indicated in Materials and Methods. The normalized genome coverage (ngCOV) of metagenome-assembled genomes (MAGs) representing methanotrophs (one genome) and methanogens (four genomes) is displayed next, followed by methane production rates in μmol of methane wet sediment g^–1^ d^–1^ as measured in triplicate methanogenic incubations, in which error bars are generally smaller than black circles. Sulfate and methane porewater concentrations are expressed in mmol L^–1^. The arrow to the right indicates decreasing bottom water (BW) oxygen concentrations from Site 3 to 7. cmbsf; centimeters below the seafloor.

Sites 5 and 7 showed particularly high potential methane production rates (up to 2.3 ± 0.3 and 3.3 ± 0.4 μmol methane g^–1^ d^–1^ respectively at 2 cm depth). By contrast, at Site 3, potential methane production rates did not exceed 0.22 ± 0.006 μmol methane g^–1^ d^–1^ at 18 cm (Figure 4 and Table S1). To examine the microbial diversity and metabolic potential, sediments were subjected to DNA extractions and high-resolution 16S rRNA gene sequencing, with selected samples also used for metagenomic sequencing. Archaeal 16S rRNA gene sequences were used to generate amplicon sequence variants (ASVs), which were clustered at the family level for relative abundance visualization (Figure 4). *Methanoregulaceae* and *Methanosaetaceae* represent the two most abundant putative methanogenic families. While *Methanoregulaceae* had the highest relative abundances of 16% in Site 3 at 34 cm, 38% in Site 5 at 50 cm, and 34% in Site 7 at 30 cm, *Methanosaetaceae* reached 11% in Site 3 at 19 cm, 20% in Site 5 at 42 cm, and 24% in Site 7 at 38 cm. Other identified putative methanogenic families within *Euryarchaeota* included *Methanosarcinaceae*, *Methanofastidiosaceae*, *Methanomicrobiaceae*, *Methanospirillaceae*, *Methanobacteriaceae*, and *Methanocorpusculaceae*, poorly resolved *Methanocellales* and *Methanomicrobiales* families, *Methanocellaceae*, and *Methanothermobacteriaceae*, and the putative methanotroph family *Methanoperedenaceae*. Within the phylum *Verstraetearchaeota*, the putative methanogenic families *Methanomethyliaceae* and *Methanomethylophilaceae* were identified, and, within the phylum *Thermoplasmatota*, *Methanomassiliicoccaceae*. An ANME-2a-2b family had the highest relative abundances, among archaeal sequences, of 32% at 2.5 cm depth at Site 3, 12% at 26 cm in Site 5, and 1% at 50 cm depth in Site 7. The putative ammonium-oxidizing *Crenarchaeota* family *Nitrosopumilaceae* reached 70% relative abundance in Site 3 at 1.25 cm and was minor in the other two sites. Of 7192 archaeal ASVs, 71% could not be classified at the family level, and their summed relative abundances varied between 22 and 86%. However, only 6% of archaeal ASVs could not be classified at the phylum level. The large proportion of unclassified archaeal ASVs at the family level could indicate taxonomic novelty or, alternatively, overestimation of biodiversity, a common limitation of 16S-based studies.^69^

In total, 144 metagenome-assembled genomes (MAGs) of high (>90% complete, <5% contaminated) and medium quality (>50% complete, <10% contaminated) were reconstructed from 12 coassembled samples and screened for methane metabolism marker genes. No particulate or soluble methane monooxygenase-encoding genes, which are diagnostic of aerobic methane oxidation potential, were found in binned and unbinned contigs. Five genes encoding a methyl-coenzyme M reductase alpha subunit (*mcrA*) related to methane production were identified in the following four genomes. MAG 009 Methanoregulaceae had potential for hydrogenotrophic methanogenesis, and MAG 010 Methanosarcinaceae had potential for methanogenesis from H_2_ and CO_2_, formate, acetate (*acss*), H_2_ and methanol (*mtaA*), and H_2_ and mono-(*mtmBC*) and trimethylamine (*mttC*), but contained two *mcrA* genes. While MAG 015 Methanomassiliicoccales had potential for methanogenesis from H_2_ and methanol (*mtaA*), MAG 016 Methanomassiliicoccales had potential for methanogenesis from H_2_ and methanol (*mtaA*), di(*mtbC*), and trimethylamine (*mttC*). We also identified an unbinned *mcrA* sequence with a best blastp hit to *Candidatus* Methanofastidiosum methylthiophilus (KYC53403.1 NCBI accession number), which has been proposed to perform methanogenesis from methanethiol, dimethylsulfide, 3-methylmercaptopropionate, and 3-mercaptopropionate.^70^

We used NMR values of genes and normalized genome coverage (ngCOV) of MAGs as a qualitative proxy for microbial abundances with the sole purpose of comparisons between samples in this study and interpret these data strictly in the context of sediment biogeochemistry and microbial activity rates. NMR values of *mcrA* genes indicated that MAG 010 Methanosarcinaceae, MAG 015 Methanomassiliicoccales, and MAG 009 Methanoregulaceae accounted for the most significant methanogen *mcrA* NMR changes across sites (Figure S2), with highest NMR values in Site 5, then Site 7, and lowest in Site 3. The summed normalized genome coverage (ngCOV) of four methanogen MAGs was highest at Site 5 (3.6× at 34 cm), followed by Site 3 (3× at 22 cm) and Site 7 (2.6× at 22 cm, Figure 4).

Putative methanogen abundances (hypothesized from MAG coverages and 16S rRNA gene-based relative abundances) and potential methane production rates have contrasting profiles and do not positively correlate (Figure 4). An explanation for these results is that sequencing data do not reflect a potentially higher methanogen biomass or activity in surface sediments (0–10 cm). Alternatively, larger pools of labile organic substrates generated from organic matter degradation could be available in surface sediments (as observed in other aquatic ecosystems^71^), which are depleted at depth, resulting in decreasing potential methane production rates in deeper sediment layers. This might become apparent at the gene expression level, which could correlate with methane production activity, which is potentially detectable in future metatranscriptomic studies. In Site 3, where sulfate was detected until ca. 30 cm, sulfate reduction-driven competitive inhibition of methanogenesis was expected,^72^ and low potential methane production rates at this site indicate that this expectation was fulfilled despite the relatively high MAG coverages and 16S-based relative abundances of methanogens. In Sites 5 and 7, high potential methane production rates in surface sediments might also reflect larger pools of labile organic carbon and the rapid depletion of sulfate. These are conditions that could favor methanogens at potentially lower abundances in top sediments to be more active than in deeper sediments, where they could be more abundant but have less substrate availability. Additionally, the observation that the highest potential methane production rates were measured in surface sediments concomitant with relatively high sulfate concentrations suggests cryptic methane cycling, in which methane is consumed as soon as it is produced within the SMTZ, detectable via radiotracer or stable isotope studies.^73^ Methanogenesis from noncompetitive substrates is supported by our data since genomic potential for methanol and methylamine-driven methanogenesis was identified in three out of four methanogen MAGs. This could partially fuel AOM in Site 5, where the ANME-2 MAG coverage was significant (3.85×) within the SMTZ. Methylotrophic methanogenesis has been previously implicated in cryptic methane cycling in similar ecosystems.^74,75^ Alternatively, competitive methanogenesis could coexist with sulfate reduction, as previously observed in estuarine systems, potentially due to the abundance of substrates.^76^ This is also supported by our data, given the relative abundances of sequences affiliated with *Methanoregulaceae* and *Methanosaetaceae*.

### Anaerobic Methane-Oxidizing Archaeon Constitutes the Benthic Methane Biofilter

**Figure 5:**
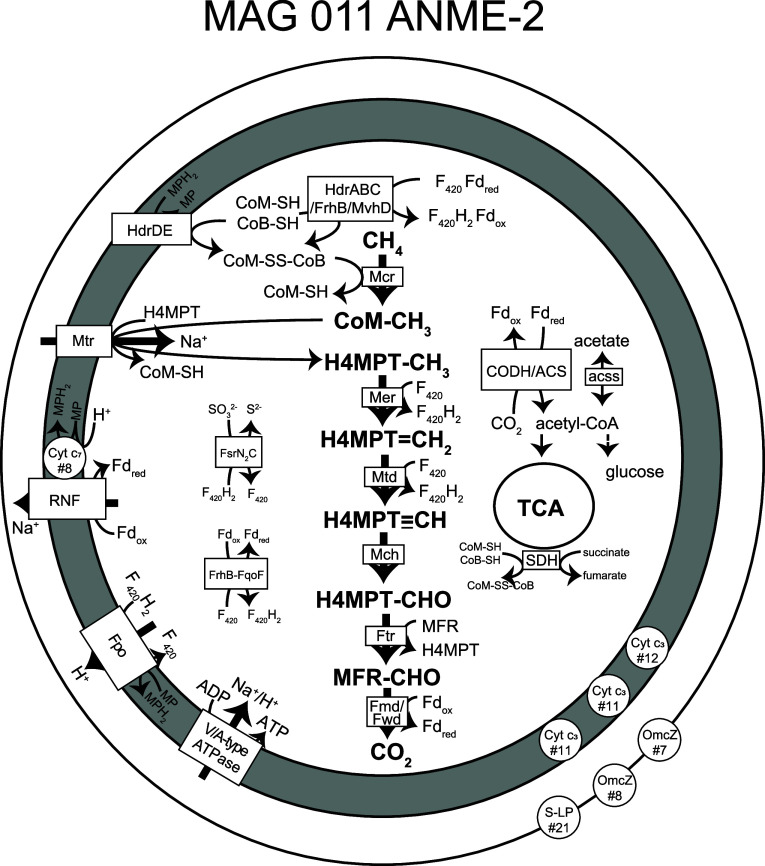
Metabolic reconstruction of MAG 011 ANME-2 based on loci specified in Table S3. The gray area represents the pseudoperiplasm, and the outermost circle represents the S-layer. Numbers that follow # indicate the number of heme-binding motifs. Abbreviations are as follows: F_420_, coenzyme F_420_; Fd, ferredoxin; CoM, coenzyme M; CoB, coenzyme B; MP, methanophenazine; HdrABC, cytoplasmic heterodisulfide reductase; Frh, F_420_-reducing hydrogenase; MvhD, methyl viologen-reducing hydrogenase subunit D; HdrDE, periplasmic heterodisulfide reductase; Mcr, methyl-coenzyme M reductase; H_4_MPT, tetrahydromethanopterin, Mtr, formylmethanofuran-H_4_MPT N-formyltransferase; Mer, F_420_-dependent methylene-H_4_MPT reductase; Mtd, F_420_-dependent methylene H_4_MPT dehydrogenase; Mch, Methenyl-H_4_MPT cyclohydrolase; Ftr, formylmethanofuran: H_4_MPT formyltransferase; Fmd, molybdenum-dependent formylmethanofuran dehydrogenase; Fwd, tungsten-dependent formylmethanofuran dehydrogenase; Cyt, cytochrome; RNF, *Rhodobacter* nitrogen fixation complex; Fpo, F_420_:methanophenazine oxidoreductase; S-LP, S-layer protein; OmcZ, outer membrane cytochrome; FsrN_2_C, F_420_-dependent sulfite reductase; FrhB-FqoF, hypothetical F_420_- and Fd-oxidizing electron-confurcating hydrogenase; CODH/ACS, carbon monoxide dehydrogenase/acetyl-CoA synthase complex EC: 1.2.7.4/2.3.1.169); acss, acetyl-CoA synthetase (EC: 6.2.1.1); TCA, tricarboxylic acid cycle; SDH, succinate dehydrogenase.

Based on phylogenetic analyses, MAG 011, affiliated with the archaeal order *Methanosarcinales*, was identified as representative of an ANME-2 archaeon (Figure S3). This was the only detected genome representing a methane-oxidizing microorganism in our sequencing data sets, indicating that the benthic methane biofilter (the biological process of methane removal in sediments) was potentially constituted of a single organism. MAG 11 had the highest ngCOV at Site 5 (9.5× at 22 cm) below the SMTZ (Figure 4), but reached only 0.3× at Site 3 at 2 cm and 0.06× at Site 7 at 2 cm. This genome had 66% average amino acid identity to genome MZXQ01 (NCBI accession number), which represents an ANME-2b archaeon obtained from sediment of the Hydrate Ridge North methane seep^77^ classified as *Ca*. *Methanomarinus* sp. nov. 1^19^ (Figure S4). MAG 011 was 98.4% complete and 2% contaminated and had potential for a full reverse methanogenesis pathway as well as acetate production or assimilation via an acetyl-CoA synthase (*acss*) gene (Figure 5 and Table S4). MAG 011 was further analyzed in detail to elucidate its metabolic potential. Genes encoding proteins involved in the reverse methanogenesis pathway were mostly present as single copy (Table S4), with a few exceptions: (i) *hdrABC* were present in three copies, (ii) a second copy of *mtrAH* was downstream of *mtrX*, while *mtrEDCBAFGH* subunits were present in a separate contig, (iii) three copies of the gene encoding the formylmethanofuran-tetrahydromethanopterin N-formyltransferase (*ftr*) were present, and (iv) both molybdenum- and tungsten-dependent formylmethanofuran dehydrogenases were present (*fmdCABDE* and *fwdGBDC*), with *fwdC* present as a separate single subunit and also as a two-copy *fwdC* gene fusion (Table S4).

Nine candidate genes encoding electron-carrying ferredoxins were identified, as well as several FrhB/FdhB/FpoF paralogues (Table S4). A single subunit *fpoF* could be part of the F_420_H_2_ dehydrogenase complex *fpoDCBAONMLKJ1J2IH*, and an *frhB*-*fqoF* (K00441, K22162) gene fusion could encode a protein to couple ferredoxin oxidation to F_420_H_2_ production, as potential Fpo/Fqo-dependent ferredoxin oxidation pathways.^19^ Moreover, three other genes with homology to FrhB of *Candidatus* Methanoperedens nitroreducens strain BLZ1^78^ were found: (i) the first as a single subunit, (ii) the second immediately upstream of *mvhD*, *hdrA2*, another *mvhD*, then *hdrABCC*, and (iii) the third as 2x*frhB*-*fsrC* fusion (K00441–K00441–K21816). The genes *fsrNC* encode an F_420_-dependent sulfite reductase in *Methanocaldococcus jannaschii*(79) (EC: 1.8.98.3), which detoxifies sulfite while reducing it to sulfide. Furthermore, the succinate dehydrogenase membrane subunits *sdhCD* were absent, while *sdhAB* were present and *sdhB* was fused with *tfrB* (K00239, K00240–K18210), which encodes the CoM/CoB-fumarate reductase subunit B (EC: 1.3.4.1) characterized in *Methanobacterium thermoautotrophicum* strain Marburg.^80^ In *M. thermoautotrophicum*, TfrA harbors FAD-binding motifs and the catalytic site for fumarate reduction, while TfrB harbors one [2Fe–2S] cluster, two [4Fe–4S] clusters, and the catalytic site for CoM–S–H and CoB–S–H oxidation. Therefore, we hypothesize that in ANME-2 represented by MAG 011, electrons from succinate oxidation could be used to generate heterodisulfide for the first step in methane oxidation instead of flowing to the electron transport chain. Finally, a complete Rnf complex, involved in ferredoxin recycling and proton gradient generation in ANME,^81^ was identified, as well as a downstream c_7_ family octaheme cytochrome as previously reported in ANME-2.^82^ Other cytochromes were also identified in this genome: three c_3_-family cytochromes containing 11 or 12 heme-binding motifs, an S-layer protein containing 21 heme-binding motifs, and two FeGenie-identified outer membrane cytochrome *omcZ* sequences with 7 and 8 heme-binding motifs (Table S4), which could mediate extracellular electron transfer to a syntrophic partner or metallic terminal electron acceptor.

### Sulfide Toxicity Hinders Methane Removal by ANME

**Figure 6:**
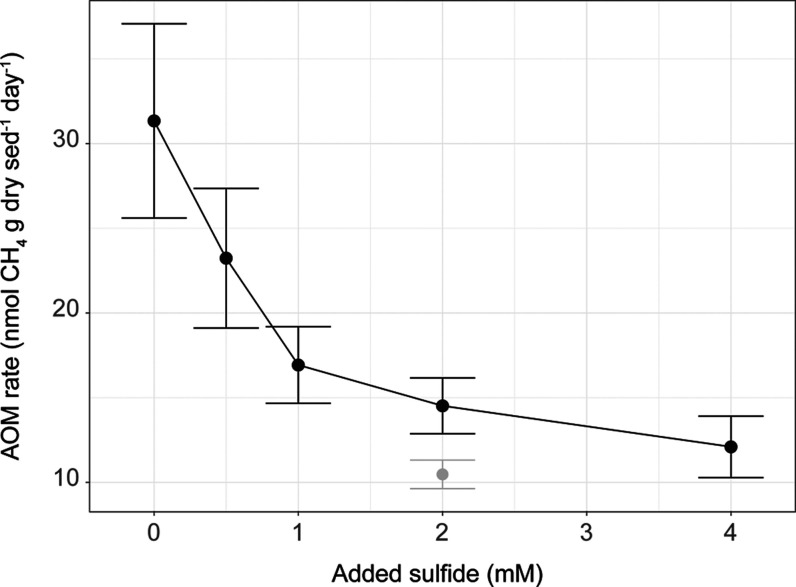
Average AOM rates measured in Site 5 sediments incubated with varying added sulfide concentrations (0, 0.5, 1, 2, or 4 mM). While black circles indicate that samples were preincubated with ^13^C-methane only, the gray circle indicates that samples were preincubated with ^13^C-methane and 2 mM of sulfide. Error bars provide the standard deviations across three biological replicates.

Based on the highest ngCOV of ANME-2 MAG 011, we selected sediments from Site 5 at the depth interval of 8–28 cm to experimentally test the hypothesis that sulfide inhibits AOM activity in these sediments (Figure 6 and Table S2). We preincubated sediments with ^13^C-methane until AOM activity was detected. Then, we added 0, 0.5, 1, 2, or 4 mM sulfide to triplicate incubations and monitored AOM rates as well as final sulfide concentrations. Additionally, we included one control to which we added 2 mM sulfide at the beginning of the preincubation. Average AOM rates were highest when no sulfide was added (31.3 nmol of methane g dry sediment^–1^ day^–1^), decreasing with increasing sulfide concentrations: 23.2, 16.9, 14.5, and 12.1 nmol of oxidized methane g dry sediment^–1^ day^–1^ when, respectively, 0.5, 1, 2, and 4 mM of sulfide were added to incubations (Figure 6 and Table S2). The average AOM rate in the control incubation that received 2 mM of sulfide at the beginning of the preincubation was 10.5 nmol of oxidized methane g dry sediment^–1^ day^–1^. This resulted in an average of 3.62 mM sulfide at the end of the experiment and inhibition of 67% of AOM activity relative to incubations that received no sulfide, which had an average of 0.55 mM of sulfide at the end of the experiment. In preincubated bottles with no added sulfide, additions of 0.5, 1, 2, and 4 mM of sulfide resulted, respectively, in average final sulfide concentrations of 0.8, 1.12, 1.88, and 3.41 mM, and inhibition of 26, 46, 54 and 61% of AOM activity, respectively (Figure 6 and Table S2).

Results from this experiment corroborate our key microbiological observation, the differential distribution of potential ANME-2 abundances across samples based on MAG 11 normalized genome coverages. Low coverages of MAG 11 were estimated in sediments of Site 3, in agreement with the low methane concentrations measured in situ and low potential methane production rates measured (Figure 4). At Site 5, MAG 11 coverages were the highest, within and below the SMTZ, matching abundant methane and sulfate substrates for S-AOM activity (Figure 4) and their calculated fluxes into the SMTZ (Table 3) as well as the highest potential methane production rates. However, at Site 7, where methane and sulfate were also abundant and had similar high fluxes into the SMTZ (Table 3), putative ANME-2 abundances were near zero, based on both 16S analyses and MAG 11 coverages.

We acknowledge that shallowing of the SMTZ and methanogenesis in surface sediments likely contributed to higher methane escape from Sites 5 and 7 relative to Site 3.^5^ Such high benthic fluxes of methane are in accordance with previously reported values for the Stockholm Archipelago.^32^ However, differences in the SMTZ or in methanogenesis in surface sediments could not account for the 7-fold higher benthic methane efflux in Site 7 relative to Site 5, given that these two sites had nearly identical depths and thicknesses of the SMTZ (0–10 cm) and similar potential methane production rates in top sediments (Figure 4).

Instead, our experimental results in combination with the virtual disappearance of ANME-2 sequences at Site 7, but their persistence at Site 5, suggests that the disruption of the methane biofilter likely accounted for this 7-fold difference. We infer that the putative distribution of ANME-2 at our study sites is most likely linked to high sulfide concentrations (0.5–1.2 mmol L^–1^) and especially the higher annual exposure to sulfide (0.88 mmol year^–1^) at Site 7 compared with that at Site 5 (0.39 mmol year^–1^), which could have directly caused sulfide toxicity in ANME-2 cells. We note that the relationship between sulfide and AOM rates is nonlinear (Figure 6), which could explain why the difference in sulfide exposure between the two sites (∼2-fold) does not mathematically account for the difference in benthic methane efflux (7-fold) between the two sites. In addition, we cannot rule out an effect of other (abiotic) factors such as differences in the type of organic matter and sediment composition in general between the two sites. These results agree with an early study on environmental controls on ANME abundances, which found that sulfide concentrations negatively correlated with 16S-based ANME-2 abundances while positively correlated with ANME-1 abundances, potentially due to sulfide-oxidizing bacteria co-occurring with ANME-1 but not with ANME-2.^83^ Additionally, sulfide could hamper the enzymatic activity of the F_420_-dependent sulfite reductase (Fsr) via product inhibition, leading to sulfite toxicity, as well. Sulfite is known to inhibit methanogenesis,^84^ and the Fsr enzyme, first described in *M. jannaschii*, detoxifies sulfite by reducing it to sulfide, which can then be assimilated. While sulfite toxicity generally occurs due to its intracellular reaction with proteins and sulfhydryl groups,^79,85^ in methanogens specifically, sulfite reacts in vitro with and inactivates the key methanogenic enzyme, methyl-coenzyme M reductase.^86,87^ The recent crystal structure of Fsr from *Methanothermococcus thermolithotrophicus* revealed Fsr as the simplest sulfite reductase crystallized so far, with similar traits to assimilatory sulfite reductases, having, interestingly, a higher preference for nitrite (apparent Km of 2.5 μM) over sulfite (apparent Km of 15.6 μM).^88^

F_420_-dependent sulfite reductases have been found to be highly abundant sulfur metabolism proteins in an ANME-2 metaproteomics study,^77^ which also reported the inhibition of AOM activity by ANME-2a/2c in methane seep sediments incubated with 1 mmol L^–1^ sulfite, 1 mmol L^–1^ polythionate, and 0.25 mmol L^–1^ polysulfide. The authors concluded that the role of F_420_-dependent sulfite reductases in ANME-2 is more likely sulfite detoxification rather than sulfur assimilation, which could occur via several other ANME-2 enzymes.^77^ Furthermore, while methanogens are known to withstand 3–5 mmol L^–1^ sulfide levels and assimilate sulfur from sulfide,^89^ ANME archaea have been reported to be inhibited by 3–4 mmol L^–1^ sulfide under the low sulfate concentrations that we measured in our study (4 mmol L^–1^ range), but not under high (21 mmol L^–1^) sulfate concentrations.^90^ Our experimental results (Figure 6) provide further evidence for a dose-dependent, sulfide-driven inhibition of AOM activity under ∼4 mmol of L^–1^ sulfate.

MAG 11 ANME-2 had several genes encoding multiheme *c*-type cytochromes (Figure 5) including S-layer proteins, lowly expressed in Fe-AOM-performing ANME-2d,^25^ and OmcZ-like proteins suggested as the ANME mechanism for extracellular electron transfer,^19^ which could be used for electron transfer to a sulfate-reducing partner or to metal oxides. These multiheme *c*-type cytochromes identified in ANME might be targets of sulfite toxicity.^91^

These previous studies suggest that a threshold sulfide concentration, potentially dependent on sulfate concentrations, might exert thermodynamic and toxicity controls on AOM activity. Our results match these previous observations but also indicate that sulfide exposure (total sulfide in mmol year^–1^), which differed more between Sites 5 and 7 than sulfide concentrations (mmol L^–1^ at the time of sampling), could play a role in the lower putative ANME-2 abundances at Site 7 (Figure 4). Overall, these results support our experimental, biogeochemical and metagenomic evidence for the proposed mechanism of sulfide toxicity as a key control on putative ANME-2 abundances and activity in coastal sediments, suggesting that the expansion of euxinia in coastal areas^92^ might increase benthic methane release into the water column and potentially coastal methane emissions to the atmosphere. This is particularly relevant for relatively shallow coastal sites such as Site 7 in the Stockholm Archipelago. Genes encoding F_420_-dependent sulfite reductases have been found in ANME-1, ANME-2, and ANME-3 genomes,^19^ suggesting that sulfide-driven sulfite toxicity may be a commonly encountered environmental pressure by ANME and, therefore, a widespread control on AOM activity.

In conclusion, our data suggest that ANME-2 archaea might be able to compensate for methane increases under hypoxic conditions but are unable to thrive under euxinic conditions because of sulfide-driven toxicity. This disruption of the methane biofilter results in increased benthic methane release into the water column in coastal ecosystems severely impacted by eutrophication and bottom water deoxygenation. Further studies should investigate if increased methane concentrations in euxinic waters result in increased emissions of methane to the atmosphere. Moreover, future studies are required to characterize the methane-oxidizing activity of ANME archaea under changing bottom water redox conditions as well as the metabolism and terminal electron acceptors utilized.

## Supporting Information Available

Bottom water redox conditions, salinity, and temperature from 1998 until 2018 in the three sites investigated in this study presented both as a summary of data as well as for individual sites; heat map of mcrA normalized mapped reads across sites and depths; UBCG tree built with 92 concatenated core archaeal genes of reference ANME genomes (as indicated by NCBI accession numbers) and the ANME-2 genome from this study; average amino acid identity (AAI) matrix with pairwise comparisons among genomes; and Methods (PDF)
Calculations of potential methane production rates in sediment incubations (XLSX)
Calculations of anaerobic oxidation of methane rates and sulfide concentrations in experimental sediment incubations (XLSX)
Pore water and sediment geochemical data presented in Figures 1 and 2, ^210^Pb data, and sulfide exposure calculations (XLSX)
List of MAG 011 ANME-2 genes and corresponding loci identified in this study (XLSX)
