# Supplementary material for: Sulfide Toxicity as Key Control on Anaerobic Oxidation of Methane in Eutrophic Coastal Sediments
Source: Environ Sci Technol. 2024 Jun 18;58(26):11421–35. doi: 10.1021/acs.est.3c10418 (PMC11223495; doi:10.1021/acs.est.3c10418)
Supplement: Supplementary file 1 — es3c10418_si_001.pdf [file es3c10418_si_001.pdf]

Supporting information of the manuscript entitled "Sulfide toxicity as key control on anaerobic oxidation of methane in eutrophic coastal sediments"

Paula Dalcin Martins[1,2*], João P. R. C. de Monlevad[1], Maider J. Echeveste Medrano[1], Wytze K. Lenstra[1,3], Anna J. Wallenius[1], Martijn Hermans[3,4], Caroline P. Slomp[1,3], Cornelia U. Welte[1], Mike S. M. Jetten[1], Niels A.G.M. van Helmond[1,3]

[1] Department of Microbiology, Radboud Institute for Biological and Environmental Sciences, Radboud University, Nijmegen, 6525 AJ, The Netherlands

[2]Department of Ecosystem and Landscape Dynamics, Institute for Biodiversity and Ecosystem Dynamics (IBED), University of Amsterdam, Amsterdam, 1098 XH, The Netherlands

[3] Department of Earth Sciences - Geochemistry, Utrecht University, Utrecht, 3584 CB, The Netherlands

[4]Baltic Sea Centre, Stockholm University, Stockholm, 114 18, Sweden

[*]Corresponding author

Summary

Number of pages: 16

Number of supplemental figures: 4

Number of supplemental tables: 4

Supplemental Figures

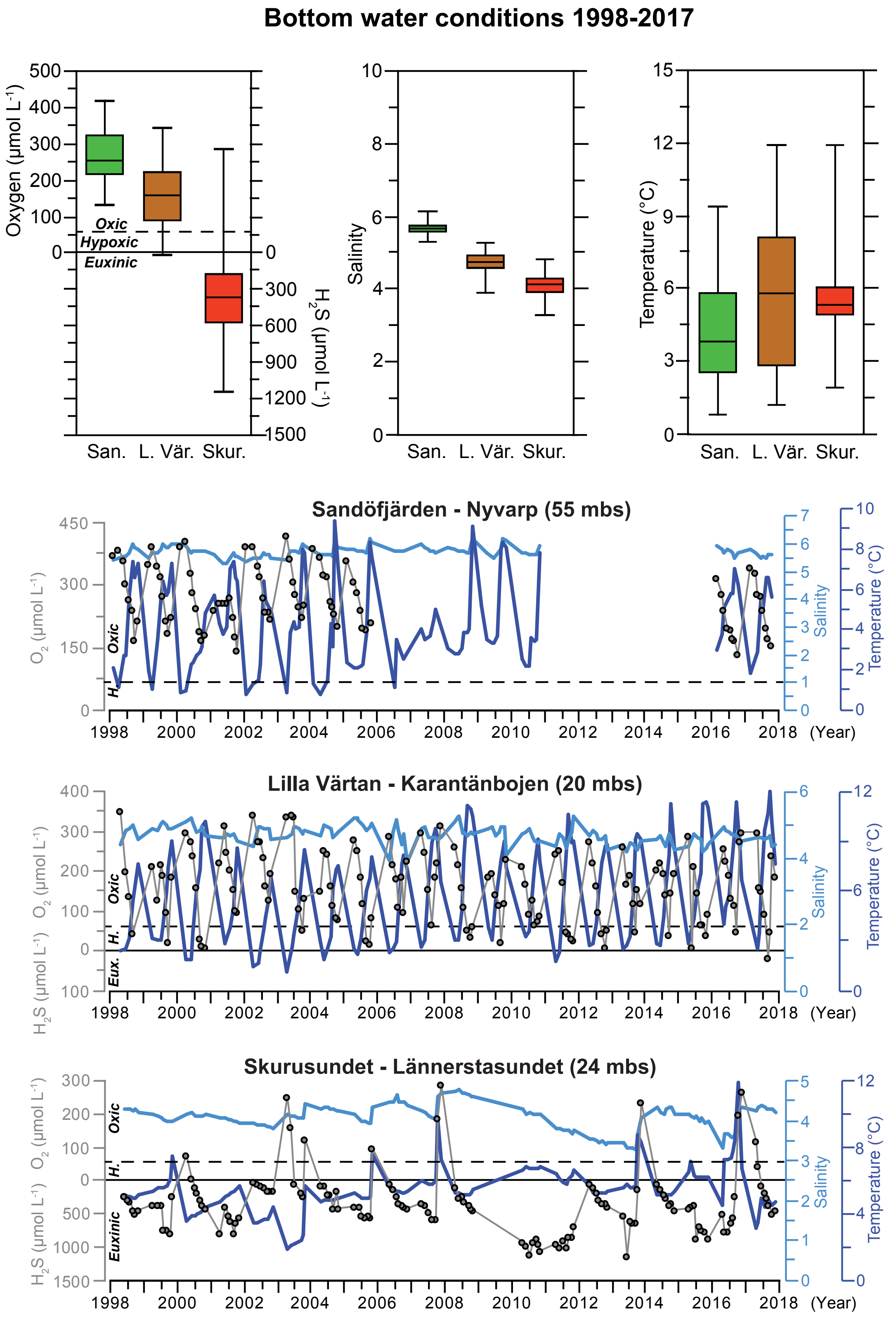

Supplementary Figure 1. Bottom water redox conditions, salinity and temperature from 1998 until 2018 in the three sites investigated in this study presented both as a summary of data as well as for individual sites. Long-term seasonal monitoring data was obtained from the Swedish Meteorological and Hydrological Institute - SMHI (https://sharkweb.smhi.se/hamta-data/).

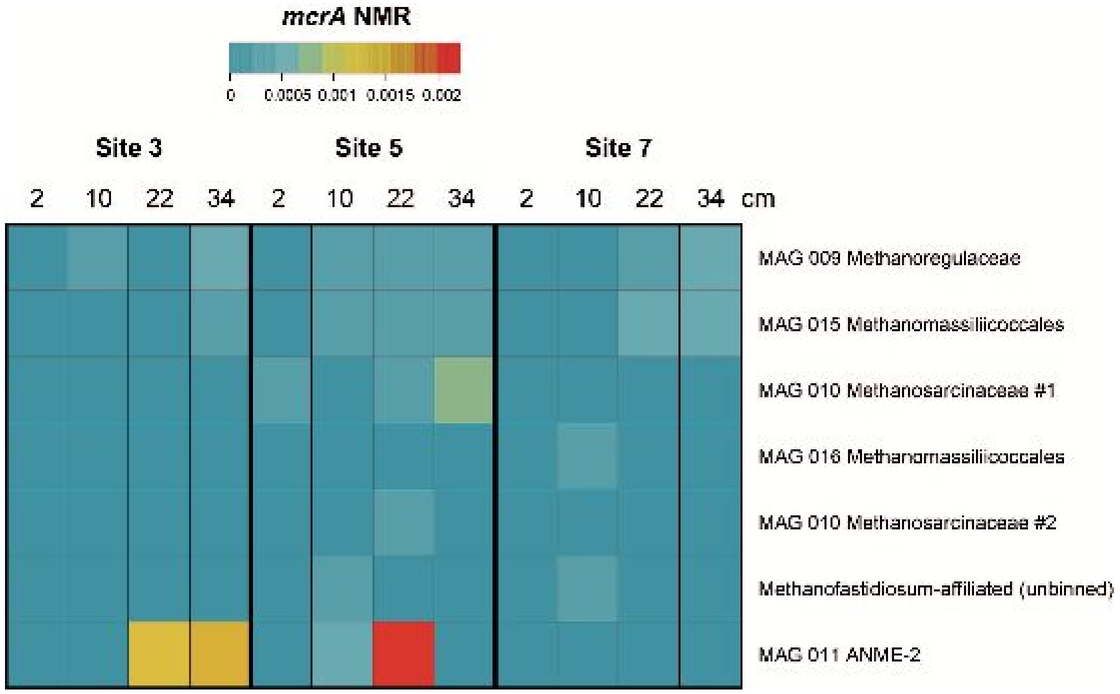

Supplemental Figure 2. Heat map of *mcrA* normalized mapped reads across sites and depths. Values were normalized to all metagenome reads.

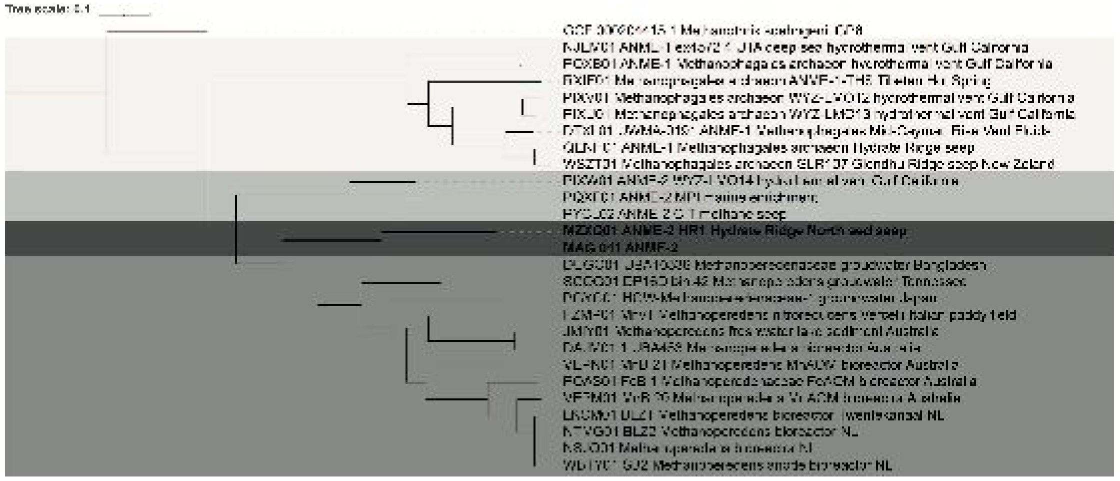

Supplemental Figure 3. UBCG tree built with 92 concatenated core archaeal genes of reference ANME genomes (as indicated by NCBI accession numbers) and the ANME-2 genome from this study. No bootstrap values were larger than 0.7.

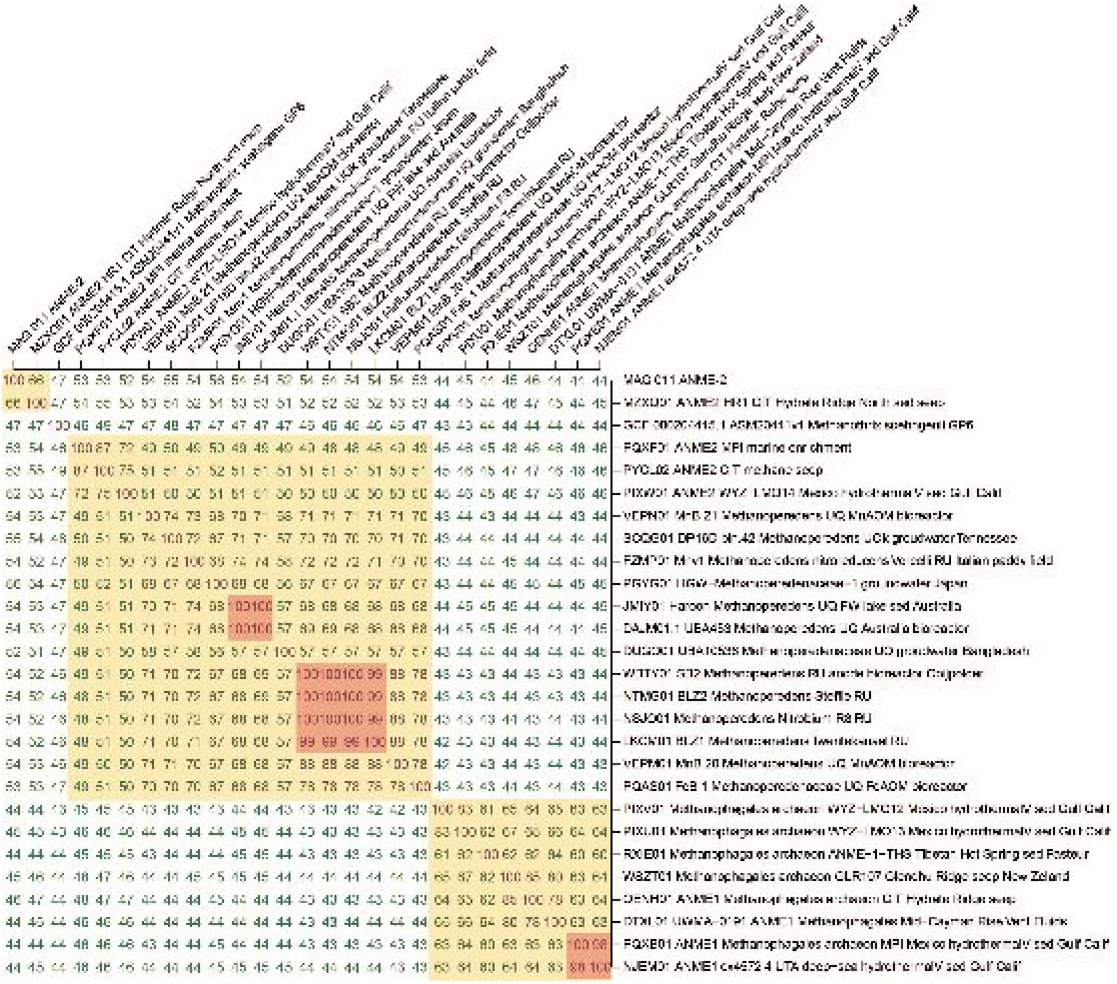

Supplemental Figure 4. Average amino acid identity (AAI) matrix with pairwise comparisons among genomes presented in Supplemental Figure 2. All prodigal-predicted protein sequences from each genome were used in AAI calculations.

Supplemental Table 1 (excel spreadsheet). Calculations of potential methane production rates in sediment incubations. Average rates and standard deviations correspond to three biological replicates (triplicate incubations). Each GC measurement was conducted in three technical replicates, which resulted in one averaged area.

Supplemental Table 2 (excel spreadsheet). Calculations of anaerobic oxidation of methane rates and sulfide concentrations in experimental sediment incubations.

Supplemental Table 3 (excel spreadsheet). Pore water and sediment geochemical data

presented in Figure 1 and Figure 2, ^{210}Pb data and sulfide exposure calculations.

Supplemental Table 4 (excel spreadsheet). List of MAG 011 ANME-2 genes and corresponding loci identified in this study.

Supplemental Materials and Methods

High-resolution depth profiling

High-resolution depth (50 µm) profiles of oxygen and pH in the overlying water and surface sediment were obtained from intact sediment cores, using microelectrodes operated by a motorized micromanipulator (Unisense A.S., Denmark). Oxygen was measured immediately, and pH was measured within 1h of core retrieval. For the oxygen microelectrode, a two-point calibration was applied (100% oxygen saturated and nitrogen purged bottom water) using a calibration vessel (Unisense A.S., Denmark, CAL300). For the calibration of the pH microelectrode, three NBS standards (pH 4, 7 and 10) were used. Then, a TRIS buffer was applied for the pH to correct for signal drift induced by salinity effects[1,2].

Collection of porewater and sediment samples for geochemical analysis

To determine porewater methane (CH_4) concentrations, samples of the bottom water and sediment were taken with cutoff 10 mL syringes via predrilled holes in the core liner with a depth spacing of 2.5 cm directly after coring. Subsequently, 10 mL of sample was transferred into a 65 mL serum bottle filled with saturated salt solution. Finally, the bottles were stoppered, capped and stored upside down until analysis. For further porewater analysis and collection of anoxic sediments, one core was sliced on board the ship in a N_2-filled portable glove bag (Sekuroka 940x940x640mm; Glas-Col, USA). Prior to anoxic sectioning of the cores, two samples were taken from the overlying water using a 20 mL syringe equipped with a three-way valve. Subsequently, cores were sliced at a resolution of 0.5 cm (0-2 cm), 1 cm (2-10 cm), 2 cm (10-20 cm), 4 cm (20-40 cm) and 5 cm until the bottom of the core. Sediment was collected in 50 mL centrifuge tubes and centrifuged at 3500 rpm for 20 minutes to extract porewater. Bottom

and porewater samples were filtered over 0.45 µm filters in a second N_2-filled portable glove bag (Sekuroka 690x690x380mm; Glas-Col, USA). Subsamples were taken for (1) sulfide - 0.5 mL of porewater was added to 2 mL 2% zinc acetate and stored at 4°C; (2) dissolved iron (Fe; assumed to be Fe^{2+}) and manganese (Mn) - 1 mL of porewater was acidified with 10 µL 30% suprapur HCl and stored at 4°C; (3) sulfate - 0.5 mL sample was collected and stored at 4°C; (4) ammonium - 1 mL sample was collected; and (5) nitrate and nitrite - 1 mL sample was collected, the latter two were stored at -20°C. The residual anoxic sediment after porewater subsampling was stored in N_2-flushed gas-tight aluminum bags at -20°C until further analysis. Samples from the core that was sliced under ambient atmospheric conditions were dried in an oven (~1 week at 60°C) to determine the sediment water content. The porosity of the sediment was then calculated assuming a solid phase density of 2.65 g cm^{-3} [3,4] and a constant density with depth given the absence of lithological changes.

Pore water analysis

Pore water sulfide was determined spectrophotometrically using phenylenediamine and ferric chloride[5]. Dissolved Fe and Mn were measured by Inductively Coupled Plasma-Optimal Emission Spectroscopy (ICP-OES) with a Perkin Elmer Avio 500 (Perkin Elmer, USA). Samples were measured with a radial plasma. Argon was used as the plasma gas (10 L min^{-1}), nebulizer gas (0.75 L min^{-1}) and auxiliary gas (0.2 L min^{-1}). Nitrogen was used as the shear gas (25 L min^{-1}). The rate of the peristaltic pump was 1.5 mL min^{-1} and the radio frequency generator was set at 1500 watt. Sulfate concentrations were determined by ion chromatography (IC) with a 930 Compact IC Flex (Metrohm, Switzerland), equipped with a Metrosep A Supp 5-150/4.0 Guard column. Blanks and three different quality controls (QCs) were run five times each per series to monitor the detection limit and reproducibility. Average analytical uncertainty based on duplicates was <1%. Ammonium concentrations were determined colorimetrically using indophenol-blue[6]. Nitrate and nitrite were determined with a Gallery™ Automated Chemistry Analyzer type 861 (Thermo Fisher Scientific, USA)[7] in accordance with NEN-ISO 15923-1 guidelines (https://www.nen.nl/en/nen-iso-15923-1-2013-en-190502).

Approximated sedimentary sulfide exposure

An approximation of the exposure of the sediments, hence the microorganisms in it, to sulfide was calculated based on sedimentation rates and pore water sulfide depth profiles (Supplemental Table 3). We calculated the cumulative concentration of sulfide that the sediments are exposed to at 30 cm depth and the average sulfide exposure per year. Sedimentation rates were calculated based on ^{210}Pb data (Supplemental Table 3) as previously described[8].

**Summary**	Site 3	Site 5	Site 7
Cumulative sulfide exposure for the top 30 cm of the sediment (mmol/30 cm)	4.0	6.3	19.3
Years captured in the top 30 cm of the sediment (year)	50	16	22
Average sulfide exposure per year (mmol/year)	0.08	0.39	0.88

Sediment analysis

Total organic carbon (TOC) was determined on a subsample of the oxic fraction as previously described[9]. Briefly, about 200 to 300 mg of sample was decalcified with 1M HCl, after which the residues were dried, powdered and weighed for analysis with a Fisons Instruments NA 1500 NCS analyzer (Carlo Erba, France). TOC was calculated after correction for the weight loss upon decalcification and the salt content of the freeze-dried sediment. Analytical uncertainty, based on duplicates (n=12), was <3%.

The total sedimentary concentrations of Fe and Mn were determined by digestion of ca. 125 mg of the freeze-dried oxic subsample in a mixture of strong acids as previously described[8,10] and analyzed for their elemental composition by ICP-OES. The accuracy (recovery) for Fe and Mn was 91 and 93%, respectively. The average analytical uncertainty based on duplicates (n=14) was <2 % for both Fe and Mn. Two subsamples of the freeze-dried

anoxic sample of about 50 mg each were subjected to two different sequential extraction procedures to determine the different solid phase forms of Fe and Mn, respectively.

For Fe, a previously described sequential extraction procedure[11] was applied, separating sedimentary Fe in: (1) poorly ordered Fe(III)-oxides (e.g. ferrihydrite and lepidocrocite); (2) Fe(II)-carbonates/monosulfides, (in this setting, with abundant porewater sulfide, presumably mainly Fe-monosulfides[8]); (3) crystalline Fe(III)-oxides (e.g. goethite and hematite (4) recalcitrant Fe(II/III)-oxides, such as magnetite; and (5) Fe(II)-pyrite. The total Fe concentrations in all extraction steps was determined with the colorimetric phenanthroline method[12]. Average analytical uncertainty for all fractions, based on duplicates (n=12), was <9% for Fe.

For Mn, a previously described procedure[10] was applied, separating sedimentary Mn in: (1) poorly ordered Mn(III/IV)-oxides (e.g. birnessite and pyrolusite); (2) Mn(II)-carbonates (rhodochrosite); (3 & 4) crystalline Mn(II/III) oxides, and (5) Mn associated with pyrite. The total Mn concentration in extraction steps (1), (2) and (5) was determined by ICP-OES. Steps (3) and (4) were not further analyzed for this study because these fractions generally represented only a minor fraction of the total Mn that was extracted in similar coastal sediments[10]. Analytical uncertainty, based on duplicates (n=10), was <5% for Mn.

DNA extractions, amplicon sequencing and 16S rRNA gene analyses

Sediments for DNA sequencing were immediately frozen at -20ºC after on board core slicing under nitrogen atmosphere and were stored at -20ºC for four months until thawing at room temperature for DNA extractions. DNA was extracted from 73 sediment samples retrieved from three cores in total, one core per site, with a depth resolution of 0.5 cm for the top 2 cm, 1 cm until 10 cm depth, 2 cm until 20 cm depth, and 4 cm below that. DNA extractions were performed with the DNeasy Power Soil Kit (Qiagen, Germany) according to the manufacturer's instructions with two modifications. For the bead-beating step, a TissueLyser LT (Qiagen, Germany) was used for 10 minutes at 50 Hz, and DNA was eluted in 30 μL of autoclaved ultrapure water (Milli-Q Reference Water Purification System, Merck & Co., USA).

DNA was quantified with a Qubit 2.0 Fluorometer (Thermo Fisher Scientific, USA). Amplicon sequencing was conducted by Macrogen Europe BV (Amsterdam, Netherlands) on an Illumina MiSeq platform using the MiSeq Reagent Kit v3, producing 2x300bp in paired-end reads. For the identification of archaea, the chosen primers were Arch349F (GYGCASCAGKCGMGAAW) and Arch806R (GGACTACVSGGGTATCTAAT)[13]. 16S rRNA gene sequencing data was processed on RStudio v1.3.959 and R v3.6.3 with the following packages: DADA2[14] v1.9.0, phyloseq[15] v1.32.0, vegan[16] v2.5-6, DESeq2[17] v1.28.1, and ggplot2[18] v3.3.5. Briefly, primers were removed with cutadapt[19] using the options -g, -G, and --discard-untrimmed. The DADA2 pipeline[20] was then used to trim forward reads at 270 nt and reverse reads at 240 nt based on quality plots (Q>20). Low quality and contaminant sequences were discarded. After error models were built, sequences were dereplicated and amplicon sequence variants (ASVs) were inferred. Forward and reverse reads were merged and chimeras were discarded. Taxonomy was assigned using the Silva non-redundant training set v138 downloaded from https://zenodo.org/record/3731176#.XoV8D4gzZaQ. ASV counts and taxonomy tables were then used for data analyses with phyloseq. ASVs were clustered by taxa and relative abundance was calculated and plotted with ggplot2.

Metagenomic sequencing and data analyses

DNA was extracted and quantified as abovementioned from four homogenized sediment samples from each of the three sites with a depth resolution of 4 cm: 0-4 cm, 9-12 cm, 21-24 cm, and 33-36 cm. These 12 samples were sequenced by Macrogen Europe BV (Amsterdam, Netherlands) using the TruSeq Nano DNA library with an insert size of 350bp on an Illumina NovaSeq6000 platform, producing 2x151bp paired-end reads (5 Gbp per sample). Read quality was assessed with FASTQC v0.11.8 before and after quality-trimming, adapter removal and contaminant filtering, performed with BBDuk (BBTools v38.75). Trimmed reads were co-assembled *de novo* using MEGAHIT v1.2.9[21] and mapped to assembled contigs using BBMap (BBTools v38.75). Sequence mapping files were handled and converted using SAMtools v1.10. Contigs at least 1000 bp-long were used for binning with CONCOCT v1.1.0[22],

MaxBin2 v2.2.7[23], and MetaBAT2 v2.15 using default parameters[24]. Resulting metagenome-assembled genomes (MAGs) were dereplicated with DAS Tool v1.1.2[25], manually curated, and taxonomically classified with GTDB-Tk v1.3.0 release 95[26]. MAG completeness and contamination was estimated with CheckM v1.1.2[27].

MAGs were annotated with DRAM v1.0[28] with default options, except -min_contig_size 2500 bp for MAGs and 5000 bp for unbinned contigs, and genes of interest were searched in annotation files. Additionally, methyl-coenzyme M reductase alpha subunit-encoding *mcrA* genes were also searched with the HMM PF02745.15 MCR_alpha_N and iron metabolism genes were searched with FeGenie[29] v1.0. Only high and medium quality MAGs (>50% complete and less than 10% contaminated) were included in genome-centric analyses, and the entire dataset (binned and unbinned contigs) was considered in gene-centric analyses. For phylogenetic trees, sequences were aligned with muscle v3.8.31 [30], alignment columns were stripped with trimAl v1.4.rev22[31] using the option -gappyout, and trees were built with FastTree v2.1.10[32] or UBCG v3.0[33]. Average amino acid identity between selected genomes was calculated using the Konstantinidis Lab tool (http://enve-omics.ce.gatech.edu/g-matrix/index). For this, genomes were gene called with Prodigal v2.6.3[34], and amino acid fasta files were used as input. The abundance of MAGs was inferred from normalized genome coverage (ngCOV), that is, MAG coverage normalized to total metagenome size. Briefly, this was calculated as the total base pairs of summed metagenome reads that mapped to MAGs multiplied by 1 Gbp divided by genome length and total summed metagenome base pairs, as previously described [35]. Normalized mapped read (NMR) values for specific genes were calculated as follows: NMR = the number of mapped reads to the gene /(the length of the gene in bp*10^3)*(the total number of reads (mapped + unmapped) in the metagenome/10^6). This is similar to values of reads per kilobase of gene per million mapped reads (RPKM), except that, instead of using only the number of metagenome mapped reads as denominator, NMR uses the total number of reads in the metagenome (the sum of mapped and unmapped reads), as in ngCOV, improving normalization for metagenome size and therefore cross-sample comparisons.

Potential methane production rate measurements

Sediments for incubations to measure potential methane production rates were sliced in a N_2-filled portable glove bag and placed into sterile plastic bags (VWR International BV, Amsterdam). The resulting slices had a resolution of 4 cm and were stored anoxically into sealed aluminum bags (Gruber-Folien GmbH & Co. KG, Germany) in the dark at 4 ºC for one month from sampling collection until bottles were assembled. For this, 5 g of wet sediments were placed into 60 mL-serum bottles, and 5 mL of sulfate-free artificial seawater (ASW) medium at pH 7.5 were added to create a 1:1 diluted slurry. Bottles were degassed and sealed under argon gas at atmospheric pressure (Linde Gas Benelux). The ASW medium was adapted from a previously published study [36] to achieve a salinity of 5.3 [8] and contained, per liter, 3.418 g NaCl, 1.54 g $MgCl_2.6H_2O$, 0.097 g KCl, 0.21 g $CaCl_2.2H2O$, 0.014 g KBr, 0.0037 H_3BO_3, 0.003g $SrCl_2.6H_2O$. 0.0004 g NaF, and 0.028 g $NaHCO_3$. No trace elements or vitamins were added. From each site, six depths were incubated in triplicate at 4 °C, resulting in 54 serum bottles. Methane production was monitored via injection of 100 µl-headspace samples into an HP 5890 gas chromatograph equipped with a Porapak Q column (80/100 mesh) and flame ionization detector (Hewlett-Packard, USA) with a detection limit of <1 ppm. Each gas sample was measured in triplicate and areas were averaged.

Methane concentrations in the headspace were derived from a calibration curve (R^2>0.99). The percent of methane in the headspace was converted to molar concentration using the ideal gas law, where one mole of an ideal gas has a volume of ~24.14 L at room temperature and 1 atm. To calculate the volume (ml) of the liquid in incubation bottles, the amount of added medium (5 mL) was summed to sediment porewater volumes (calculated from dry weight measurements). Dissolved methane in the liquid was then estimated based on Bunsen solubility coefficients extrapolated from experimental data[37]. For 4˚C and a salinity of 5.3, the dimensionless solubility was estimated to be 0.0493, resulting in a Henry's law coefficient (H^{cp}) of 0.0022 mol L^{-1} atm^{-1} as calculated with equation 12 from Sander *et al.*, 2015[38]. From this, we used Henry's law ($C_{gas} = H^{cp} \times P_{gas}$) to estimate that 1 atm 100% methane at 4˚C has a solubility of 2.2 mM in seawater with a salinity of 5.3, and then liquid-dissolved methane

concentrations were proportionally adjusted to gas-phase methane concentrations. Gas-phase and liquid-phase methane amounts were summed to obtain the total amount of methane in each bottle for each time point. Potential rates of methane production were calculated using linear regression of methane measurements obtained in the first 8-9 days of sediment incubation. All calculations and measurements are provided as Supplemental Information.

Sulfide toxicity experiment and AOM rate measurements

Sediments for incubations to measure AOM rates under different sulfide concentrations were retrieved from previously stored, anoxically sealed aluminum bags kept in the dark at 4ºC for approximately two years after sample collection until bottles were assembled. For these incubations, Site 5 was selected due to highest coverages of the methanotroph genome (MAG 011 ANME-2), and samples were combined and homogenized, resulting in a mixture spanning the depth of 8 to 28 cm. Five grams of wet sediments were placed into 60 mL-serum bottles and mixed with 5 mL of a solution containing $MgSO_4$ 8mM and 0.5% NaCl, to achieve a final concentration of 4 mM sulfate and a pH buffered to 7.05. Bottles were degassed with argon gas and a headspace at 0.5 bar of overpressure was created, containing also 0.5% of N_2, 0.5% CO_2), and 20% $^{13}CH_4$. To one set of triplicate bottles, sulfide was added to a final concentration of 2 mM, while the other bottles did not received sulfide. After 13 weeks of incubation in the dark at 4ºC, AOM was confirmed by detection of $^{13}CO_2$ production (data not shown). Then, sulfide was added to the remaining bottles, in triplicate incubations, targeting final concentrations of 0, 0.5, 1, 2 and 4 mM. To monitor $^{13}CO_2$ production, bottle pressure was monitored using a GHM 3111 Digital Pressure Meter with a GMSD 2 BR – K31 sensor, and 50 µl of headspace was injected into an Agilent 6890 series gas chromatograph coupled to a mass spectrometer equipped with a Porapak Q column heated at 80°C with helium as the carrier gas as previously described [39]. Liquid-dissolved $^{13}CO_2$ was estimated with the equation $\sum {}^{13}CO_2 = {}^{13}CO_{2(g)} [1 + k RT\ V_{liquid}/V_{gas} (1 + K_Z/[H^+])]$[40] and summed to headspace $^{13}CO_2$ derived from calibration curves for AOM rate calculations (Supplementary Table 4). Bottles were sampled for sulfide determination as described in the section collection of porewater and sediment samples for

geochemical analysis. For that, approximately 1 mL of slurry was anoxically withdrawn and filtered through a 0.2μm Nylon syringe filter. Approximately 300 μL of filtrate was mixed with 1.2 mL of an anoxic 2% zinc acetate solution and stored at 4 ºC until analysis. Removed liquid volumes were taken into account for AOM rate calculations. Finally, sulfide concentrations were measured as described in the section "pore water analyses".

References

(1) Dickson, A. G. Standard Potential of the Reaction: AgCl(s) + 12H2(g) = Ag(s) + HCl (Aq), and and the Standard Acidity Constant of the Ion HSO4− in Synthetic Sea Water from 273.15 to 318.15 K. *J Chem Thermodyn* **1990**, *22* (2), 113–127. https://doi.org/10.1016/0021-9614(90)90074-Z.

(2) Dickson, A. G.; Sabine, C. L.; Christian, J. R. *Guide to Best Practices for Ocean CO2 Measurements*; 2007.

(3) Schulz, H. D.; Zabel, M. *Marine Geochemistry*, 2nd ed.; Springer: Heidelberg, Germany, 2006.

(4) Burdige, D. J. *Geochemistry of Marine Sediments*; Princeton University Press: Princeton, Woodstock, 2006.

(5) Cline, J. D. Spectrophotometric Determination of Hydrogen Sulfide in Natural Waters. *Limnol Oceanogr* **1969**, *14* (3), 454–458. https://doi.org/10.4319/lo.1969.14.3.0454.

(6) Solórzano, L. Determination of Ammonia in Natural Waters by the Phenolhypochlorite Method. *Limnol Oceanogr* **1969**, *14* (5), 799–801. https://doi.org/10.4319/lo.1969.14.5.0799.

(7) Doane, T. A.; Horwáth, W. R. Spectrophotometric Determination of Nitrate with a Single Reagent. *Anal Lett* **2003**, *36* (12), 2713–2722. https://doi.org/10.1081/AL-120024647.

(8) van Helmond, N. A. G. M.; Robertson, E. K.; Conley, D. J.; Hermans, M.; Humborg, C.; Kubeneck, L. J.; Lenstra, W. K.; Slomp, C. P. Removal of Phosphorus and Nitrogen in Sediments of the Eutrophic Stockholm Archipelago, Baltic Sea. *Biogeosciences* **2020**, *17* (10), 2745–2766. https://doi.org/10.5194/bg-17-2745-2020.

(9) van Helmond, N. A. G. M.; Jilbert, T.; Slomp, C. P. Hypoxia in the Holocene Baltic Sea: Comparing Modern versus Past Intervals Using Sedimentary Trace Metals. *Chem Geol* **2018**, *493*, 478–490. https://doi.org/10.1016/j.chemgeo.2018.06.028.

(10) Lenstra, W. K.; Klomp, R.; Molema, F.; Behrends, T.; Slomp, C. P. A Sequential Extraction Procedure for Particulate Manganese and Its Application to Coastal Marine Sediments. *Chem Geol* **2021**, *584*, 120538. https://doi.org/10.1016/j.chemgeo.2021.120538.

(11) Kraal, P.; Dijkstra, N.; Behrends, T.; Slomp, C. P. Phosphorus Burial in Sediments of the Sulfidic Deep Black Sea: Key Roles for Adsorption by Calcium Carbonate and Apatite Authigenesis. *Geochim Cosmochim Acta* **2017**, *204*, 140–158. https://doi.org/10.1016/j.gca.2017.01.042.

(12) Saywell, L. G.; Cunningham, B. B. Determination of Iron: Colorimetric o-

Phenanthroline Method. *Industrial & Engineering Chemistry Analytical Edition* **1937**, *9* (2), 67–69. https://doi.org/10.1021/ac50106a005.

(13) Takai, K.; Horikoshi, K. Rapid Detection and Quantification of Members of the Archaeal Community by Quantitative PCR Using Fluorogenic Probes. *Appl Environ Microbiol* **2000**, *66* (11), 5066–5072. https://doi.org/10.1128/AEM.66.11.5066-5072.2000.

(14) Callahan, B. J.; McMurdie, P. J.; Rosen, M. J.; Han, A. W.; Johnson, A. J. A.; Holmes, S. P. DADA2: High-Resolution Sample Inference from Illumina Amplicon Data. *Nat Methods* **2016**, *13* (7), 581–583. https://doi.org/10.1038/nmeth.3869.

(15) McMurdie, P. J.; Holmes, S. Phyloseq: An R Package for Reproducible Interactive Analysis and Graphics of Microbiome Census Data. *PLoS One* **2013**, *8* (4), e61217. https://doi.org/10.1371/journal.pone.0061217.

(16) Oksanen, J.; Blanchet, F. G.; Friendly, M.; Kindt, R.; Legendre, P.; McGlinn, D.; Minchin, P. R.; O'Hara, R. B.; Simpson, G. L.; Solymos, P.; Stevens, M. H. H.; Szoecs, E.; Wagner, H. Vegan: Community Ecology Package. 2019.

(17) Love, M. I.; Huber, W.; Anders, S. Moderated Estimation of Fold Change and Dispersion for RNA-Seq Data with DESeq2. *Genome Biol* **2014**, *15* (12), 550. https://doi.org/10.1186/s13059-014-0550-8.

(18) Wickham, H. *Ggplot2: Elegant Graphics for Data Analysis*; Springer-Verlag New York, 2016.

(19) Martin, M. Cutadapt Removes Adapter Sequences from High-Throughput Sequencing Reads. *EMBnet J* **2011**, *17* (1), 10. https://doi.org/10.14806/ej.17.1.200.

(20) Lee, M. Happy Belly Bioinformatics: An Open-Source Resource Dedicated to Helping Biologists Utilize Bioinformatics. *Journal of Open Source Education* **2019**, *2* (19), 53. https://doi.org/10.21105/jose.00053.

(21) Li, D.; Luo, R.; Liu, C.-M.; Leung, C.-M.; Ting, H.-F.; Sadakane, K.; Yamashita, H.; Lam, T.-W. MEGAHIT v1.0: A Fast and Scalable Metagenome Assembler Driven by Advanced Methodologies and Community Practices. *Methods* **2016**, *102*, 3–11. https://doi.org/10.1016/j.ymeth.2016.02.020.

(22) Alneberg, J.; Bjarnason, B. S.; de Bruijn, I.; Schirmer, M.; Quick, J.; Ijaz, U. Z.; Lahti, L.; Loman, N. J.; Andersson, A. F.; Quince, C. Binning Metagenomic Contigs by Coverage and Composition. *Nat Methods* **2014**, *11* (11), 1144–1146. https://doi.org/10.1038/nmeth.3103.

(23) Wu, Y. W.; Simmons, B. A.; Singer, S. W. MaxBin 2.0: An Automated Binning Algorithm to Recover Genomes from Multiple Metagenomic Datasets. *Bioinformatics* **2015**, *32* (4), 605–607. https://doi.org/10.1093/bioinformatics/btv638.

(24) Kang, D. D.; Li, F.; Kirton, E.; Thomas, A.; Egan, R.; An, H.; Wang, Z. MetaBAT 2: An Adaptive Binning Algorithm for Robust and Efficient Genome Reconstruction from Metagenome Assemblies. *PeerJ* **2019**, *7*, e7359. https://doi.org/10.7717/peerj.7359.

(25) Sieber, C. M. K.; Probst, A. J.; Sharrar, A.; Thomas, B. C.; Hess, M.; Tringe, S. G.; Banfield, J. F. Recovery of Genomes from Metagenomes via a Dereplication, Aggregation and Scoring Strategy. *Nat Microbiol* **2018**, *3* (7), 836–843. https://doi.org/10.1038/s41564-018-0171-1.

(26) Chaumeil, P.-A.; Mussig, A. J.; Hugenholtz, P.; Parks, D. H. GTDB-Tk: A Toolkit to Classify Genomes with the Genome Taxonomy Database. *Bioinformatics* **2019**, *36* (6), 1925–1927.

https://doi.org/10.1093/bioinformatics/btz848.

(27) Parks, D. H.; Imelfort, M.; Skennerton, C. T.; Hugenholtz, P.; Tyson, G. W. CheckM: Assessing the Quality of Microbial Genomes Recovered from Isolates, Single Cells, and Metagenomes. *Genome Res* **2015**, *25* (7), 1043–1055. https://doi.org/10.1101/gr.186072.114.

(28) Shaffer, M.; Borton, M. A.; McGivern, B. B.; Zayed, A. A.; La Rosa, S. L.; Solden, L. M.; Liu, P.; Narrowe, A. B.; Rodríguez-Ramos, J.; Bolduc, B.; Gazitua, M. C.; Daly, R. A.; Smith, G. J.; Vik, D. R.; Pope, P. B.; Sullivan, M. B.; Roux, S.; Wrighton, K. C.; Gazitúa, M. C.; Daly, R. A.; Smith, G. J.; Vik, D. R.; Pope, P. B.; Sullivan, M. B.; Roux, S.; Wrighton, K. C. DRAM for Distilling Microbial Metabolism to Automate the Curation of Microbiome Function. *Nucleic Acids Res* **2020**, *48* (16), 8883–8900. https://doi.org/10.1093/nar/gkaa621.

(29) Garber, A. I.; Nealson, K. H.; Okamoto, A.; McAllister, S. M.; Chan, C. S.; Barco, R. A.; Merino, N. FeGenie: A Comprehensive Tool for the Identification of Iron Genes and Iron Gene Neighborhoods in Genome and Metagenome Assemblies. *Front Microbiol* **2020**, *11*, 37. https://doi.org/10.3389/fmicb.2020.00037.

(30) Edgar, R. C. MUSCLE: Multiple Sequence Alignment with High Accuracy and High Throughput. *Nucleic Acids Res* **2004**, *32* (5), 1792–1797. https://doi.org/10.1093/nar/gkh340.

(31) Capella-Gutierrez, S.; Silla-Martinez, J. M.; Gabaldon, T. TrimAl: A Tool for Automated Alignment Trimming in Large-Scale Phylogenetic Analyses. *Bioinformatics* **2009**, *25* (15), 1972–1973. https://doi.org/10.1093/bioinformatics/btp348.

(32) Price, M. N.; Dehal, P. S.; Arkin, A. P. FastTree 2 - Approximately Maximum-Likelihood Trees for Large Alignments. *PLoS One* **2010**, *5* (3). https://doi.org/10.1371/journal.pone.0009490.

(33) Na, S.-I.; Kim, Y. O.; Yoon, S.-H.; Ha, S.; Baek, I.; Chun, J. UBCG: Up-to-Date Bacterial Core Gene Set and Pipeline for Phylogenomic Tree Reconstruction. *Journal of Microbiology* **2018**, *56* (4), 280–285. https://doi.org/10.1007/s12275-018-8014-6.

(34) Hyatt, D.; Chen, G.-L.; LoCascio, P. F.; Land, M. L.; Larimer, F. W.; Hauser, L. J. Prodigal: Prokaryotic Gene Recognition and Translation Initiation Site Identification. *BMC Bioinformatics* **2010**, *11* (1), 119. https://doi.org/10.1186/1471-2105-11-119.

(35) Dalcin Martins, P.; Frank, J.; Mitchell, H.; Markillie, L. M.; Wilkins, M. J. Wetland Sediments Host Diverse Microbial Taxa Capable of Cycling Alcohols. *Appl Environ Microbiol* **2019**, *85* (12). https://doi.org/10.1128/AEM.00189-19.

(36) Kester, D. R.; Duedall, I. W.; Connors, D. N.; Pytkowicz, R. M. Preparation of Artificial Seawater. *Limnol Oceanogr* **1967**, *12* (1), 176–179. https://doi.org/10.4319/lo.1967.12.1.0176.

(37) Yamamoto, S.; Alcauskas, J. B.; Crozier, T. E. Solubility of Methane in Distilled Water and Seawater. *J Chem Eng Data* **1976**, *21* (1), 78–80. https://doi.org/10.1021/je60068a029.

(38) Sander, R. Compilation of Henry's Law Constants (Version 4.0) for Water as Solvent. *Atmos Chem Phys* **2015**, *15* (8), 4399–4981. https://doi.org/10.5194/acp-15-4399-2015.

(39) Dalcin Martins, P.; Jong, A.; Lenstra, W. K.; Helmond, N. A. G. M.; Slomp, C. P.; Jetten, M. S. M.; Welte, C. U.; Rasigraf, O. Enrichment of Novel

Verrucomicrobia, Bacteroidetes, and Krumholzibacteria in an Oxygen-limited Methane- and Iron-fed Bioreactor Inoculated with Bothnian Sea Sediments. *Microbiologyopen* **2021**, *10* (1). https://doi.org/10.1002/mbo3.1175.

(40) He, Z.; Wang, J.; Hu, J.; Zhang, H.; Cai, C.; Shen, J.; Xu, X.; Zheng, P.; Hu, B. Improved PCR Primers to Amplify 16S RRNA Genes from NC10 Bacteria. *Appl Microbiol Biotechnol* **2016**, *100* (11), 5099–5108. https://doi.org/10.1007/s00253-016-7477-9.
